# 4*π*-periodic Josephson supercurrent in HgTe-based topological Josephson junctions

**DOI:** 10.1038/ncomms10303

**Published:** 2016-01-21

**Authors:** J. Wiedenmann, E. Bocquillon, R. S. Deacon, S. Hartinger, O. Herrmann, T. M. Klapwijk, L. Maier, C. Ames, C. Brüne, C. Gould, A. Oiwa, K. Ishibashi, S. Tarucha, H. Buhmann, L. W. Molenkamp

**Affiliations:** 1 Physikalisches Institut (EP3), Universität Würzburg, Am Hubland, D-97074 Würzburg, Germany; 2 Advanced Device Laboratory, RIKEN, 2-1 Hirosawa, Wako-shi, Saitama 351-0198, Japan; 3 Center for Emergent Matter Science, RIKEN, 2-1 Hirosawa, Wako-shi, Saitama 351-0198, Japan; 4 Kavli Institute of Nanoscience, Faculty of Applied Sciences, Delft University of Technology, Lorentzweg 1, 2628 CJ Delft, The Netherlands; 5 Laboratory for Quantum Limited Devices, Physics Department, Moscow State Pedagogical University, 29 Malaya Pirogovskaya Moscow 119992, Russia; 6 The Institute of Scientific and Industrial Research, Osaka University 8-1 Mihogaoka, Ibaraki, Osaka 567-0047, Japan; 7 Department of Applied Physics, University of Tokyo, 7-3-1 Hongo, Bunkyo-ku, Tokyo 113-8656, Japan

## Abstract

The Josephson effect describes the generic appearance of a supercurrent in a weak link between two superconductors. Its exact physical nature deeply influences the properties of the supercurrent. In recent years, considerable efforts have focused on the coupling of superconductors to the surface states of a three-dimensional topological insulator. In such a material, an unconventional induced *p*-wave superconductivity should occur, with a doublet of topologically protected gapless Andreev bound states, whose energies vary 4*π*-periodically with the superconducting phase difference across the junction. In this article, we report the observation of an anomalous response to rf irradiation in a Josephson junction made of a HgTe weak link. The response is understood as due to a 4*π*-periodic contribution to the supercurrent, and its amplitude is compatible with the expected contribution of a gapless Andreev doublet. Our work opens the way to more elaborate experiments to investigate the induced superconductivity in a three-dimensional insulator.

## Abstract (web summary)

A material weakly linking two superconductors may itself exhibit superconductivity whilst its material properties strongly influence the nature of the supercurrent. Here, the authors identify a supercurrent with *p*-wave symmetry in such a Josephson junction made of topologically non-trivial material.

## Graphical abstract

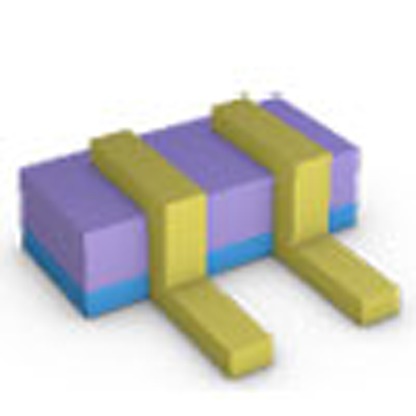

The helical nature of the topological surface states, where the spin is locked perpendicular to the momentum^1^, is predicted to give rise to exotic superconductivity when coupled to the conventional pairing potential of a *s*-type superconductor. The broken spin rotation symmetry allows the appearance of triplet *p*-wave correlations and of gapless Andreev bound states, regardless of the microscopic details of the theoretical model^2^,^3^,^4^,^5^. In a three-dimensional (3D) topological insulator (TI)-based Josephson junction (JJ), in which superconductivity is induced by the proximity effect of a *s*-wave superconductor (Fig. 1a), Andreev-bound states appear in the induced gap *Δ*_i_. They can be pictured as (Fig. 1b) a single topological Andreev doublet (depicted in blue) that occurs at transverse momentum *k*_*y*_=0 and is immune to back-scattering (thus has perfect transmission), and non-topological oblique modes (*k*_*y*_≠0, depicted in red) that are expected to have lower transmissions. The topological protection of the zero mode constitutes a superconducting analogue to Klein tunnelling^5^. As depicted in Fig. 1b, the peculiarity of this topological doublet is its 4*π*-periodicity (or equivalently a contribution *I*_4*π*_ sin *φ*/2 to the supercurrent) with respect to the superconducting phase difference *φ* across the junction^4^,^6^,^7^. The non-ambiguous observation of such gapless states is regarded as an important experimental signature of the unconventional superconductivity in TIs, but no robust evidence has been reported yet^8^,^9^,^10^,^11^,^12^,^13^. A major hindrance could be the coexistence of residual bulk conductance or of a large number of gapped conventional modes^12^,^13^. Furthermore, the finite lifetime of the positive energy branch prevents observation of a 4*π*-periodic Josephson effect in stationary measurements as various relaxation mechanisms can restore a 2*π*-periodicity for the current–phase relation (CPR)^14^,^15^,^16^,^17^. The 4*π*-periodic Josephson effect can therefore be unveiled more easily by the dynamics of the junction. To reveal the periodicity of the Josephson supercurrent, an rf driving current *I*_rf_ is added to the dc drive to induce the so-called Shapiro steps^18^. When the dynamics of a conventional JJ is phase-locked to the rf drive, steps of constant voltage appear in the *I*–*V* characteristic of the junction for voltages *V*_*n*_=*nhf*/2*e* where 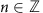 is the step index. However, for a purely 4*π*-periodic supercurrent, only a sequence of even steps should be observed. In the case of nanowires^19^, signs of the disappearance of the first step (*n*=1) have been reported and attributed to a theoretically expected topological phase transition driven by a magnetic field along the axis of the nanowire, although the topological state in the nanowires has yet to be identified in the normal transport regime. Even though it is known that other systems (JJ between *p*/*d*-wave superconductors^14^,^20^) could lead to similar effects without the need for topological protection, such effects have yet to be observed.

In this article, we study HgTe, a genuine 3D TI whose topological properties have been established independently^21^,^22^, and observe an anomalous doubled Shapiro step appearing at low frequency (equivalently a missing *n*=1 step). While several other mechanisms (non-linearities, capacitance effects, higher harmonics in the CPR^23^,^24^) are known to cause the appearance of additional subharmonic steps in the Shapiro response, to our knowledge, only the existence of a 4*π*-periodic contribution *I*_4*π*_ sin *φ*/2 in the total supercurrent can be responsible for the disappearance of odd steps^25^.

## Results

### Device characterization

**Figure 1:**
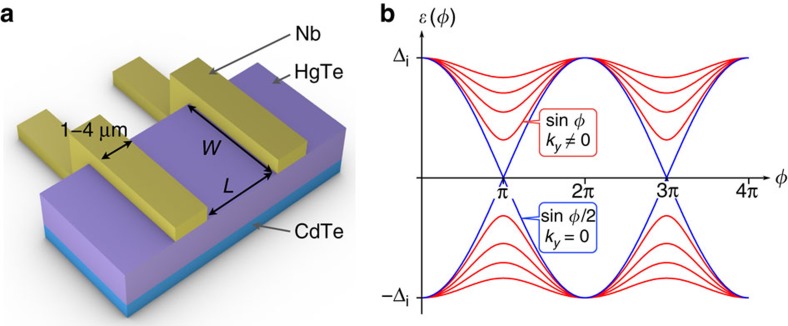
Geometry of the Josephson junction and predicted Andreev spectrum. (**a**) Artist view of the Josephson junction. Mesa stripes of HgTe (represented in mauve) are patterned on the CdTe substrate (blue), with a width *W*=2 μm. Nb contacts (in yellow) are added at the surface, with a width of 1–4 μm, separated by a variable distance *L*. (**b**) Typical energy spectra *ɛ*(*φ*) of the Andreev bound states in a 3D TI-based junction, as a function of the phase difference *φ* in the JJ, with Δ_i_. The gapless 4*π*-periodic topological mode is depicted as a blue line, corresponding to transverse momentum *k*_*y*_=0 and contributing to the 4*π*-periodic supercurrent *I*_4*π*_ sin *φ*/2. Gapped modes depicted in red correspond to *k*_*y*_≠0 and contribute to the 2*π*-periodic supercurrent *I*_2*π*_ (containing sin *φ* and higher order harmonics).

**Figure 2:**
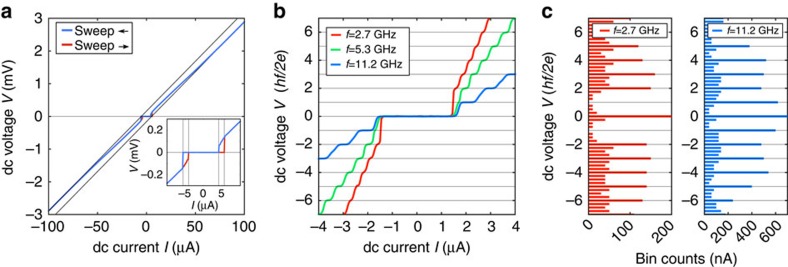
*I*–*V* curves of the *L*=150-nm junction. (**a**) *I*–*V* curve in the absence of rf irradiation for the two sweep directions, taken at base temperature *T*≃30 mK. The asymptotes (grey solid lines) do not cross the origin, emphasizing the presence of an excess current. (Inset) Detailed view of the *I*–*V* curve, that exhibits hysteresis between the upward and downward sweep direction. (**b**) Shapiro steps for three different frequencies measured at *T*≃800 mK. The plotted voltage scale is in normalized units *hf*/2*e* to highlight the formation of Shapiro steps in the *I*–*V* curve in the presence of rf irradiation. For a high-frequency *f*=11.2 GHz (blue line), all steps are clearly visible for voltages 
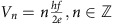
 (up to |*n*|>12, but only the first three are shown for the sake of clarity). For an intermediate frequency (*f*=5.3 GHz, blue line), the first step (*n*=1) is noticeably reduced. At low frequency (*f*=2.7 GHz, red line), the first step is fully suppressed, while all other steps remain visible. (**c**) Bar plots obtained by binning the measurement data according to voltage, for *f*=2.7 GHz and 11.2 GHz. The Shapiro steps appear as peaks in the bin counts for 
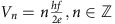
. While all steps are visible for *f*=11.2 GHz, the first Shapiro step (*n*=1) is absent at *f*=2.7 GHz.

Our devices are fabricated from coherently strained undoped HgTe layers of 65–90 nm thickness, epitaxially grown on a CdTe substrate. The band inversion of HgTe enforces the existence of topological surface states, while strain opens a gap (≃22 meV) in the bulk of the material^26^. Previous work has highlighted the high quality of the topological states in this material^9^,^21^,^22^. Quantized Hall plateaus are routinely observed, which demonstrate that transport occurs exclusively through the surface states, without any detectable parallel conductance from the bulk. The mobility and charge density, relevant for our experiments, are evaluated from a Hall bar produced separately from the same wafer as the junctions, and yield typically *μ*=1–3 × 10^4^ cm^2^ V^−1^ s^−1^, and *n*_e_=3−7 × 10^11^ cm^−2^. From these values, we extract a mean free path of 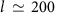 nm. The JJs are fabricated by depositing niobium contacts at the surface of a HgTe mesa, using standard sputtering and lift-off techniques (see Methods section and Supplementary Fig. 1). The geometry is shown in Fig. 1a. Each superconducting contact has a width of 1–4 μm, the HgTe weak link has a width of *W*=2 μm (corresponding to the width of the mesa stripe) and a variable length *L* ranging from 150 to 600 nm. From the electron density *n*_e_, we evaluate the number of transport modes 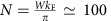. The niobium of the contacts has a critical temperature of *T*_c_≃8 K, slightly lower than that of bulk Nb (9.2 K). A typical *I*–*V* curve obtained at 30 mK is presented in Fig. 2a and exhibits hysteresis, as commonly reported^9^,^27^. We find that the critical current of devices with the same dimensions varies by about 30%, which underlines the reproducibility and quality of the fabrication process. A recurring feature in all devices is the presence of an excess current in the *I*–*V* curve (Fig. 2a, Supplementary Note 1 and Fig. 2). For high biases, the *I*–*V* curves become linear with an asymptote that does not go through the origin but is shifted towards higher currents. This excess current is understood as due to the fact that electrons in an energy window near the superconducting gap carry twice as much current due to Andreev reflections^28^,^29^. It thus illustrates the presence of Andreev reflections at both S–TI interfaces. Such an excess current has been previously observed for superconducting point-contacts^30^,^31^, but is not commonly reported in thin film structures presumably due to the presence of elastic scattering. This emphasizes the high quality and reproducibility of our devices in agreement with our previous observations^9^,^10^,^32^.

### AC response

We now turn to the study of the response of these devices to rf irradiation and highlight the existence of a 4*π*-periodic supercurrent. To this end, we focus on three devices produced from the same wafer, for which the width of the junction is set to *W*=2 μm, for nominal lengths of *L*=150, 400 and 600 nm. The experiment described below has been repeated on more than 10 devices, made out of three different wafers with similar characteristics, in three different measurement setups, all yielded similar results (Supplementary Fig 3 to 10 and Note 2).

To observe the Shapiro steps, the sample is irradiated with a radio-frequency excitation via a coaxial line, the open end of which is adjusted to be around 1 mm from the sample. In this geometry, frequencies in the range of 2–12 GHz are easily accessible, but the rf power supplied to the sample is not calibrated. Under rf irradiation, we observe the appearance of Shapiro steps in the *I*–*V* characteristic at quantized voltages *V*_*n*_=*nhf*/2*e*, where 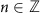 is the step index^18^. In contrast to the standard JJ response, with steps at each *n*, we find at lower frequency that the *n*=1 step is missing. To illustrate this anomalous Shapiro response of our junctions, we present three *I*–*V* curves corresponding to three different excitation frequencies in Fig. 2b (for the junction with *L*=150 nm). The applied rf power is chosen such that all curves display similar critical currents, the full range of rf power will be discussed later. For a high-frequency *f*=11.2 GHz, one typical *I*–*V* curve is plotted as a blue line (with voltage normalized to *hf*/2*e*). Several steps are clearly visible with step height *hf*/2*e*. At lower frequencies *f*=5.3 GHz (green line), higher order steps are visible but a clear reduction of the amplitude of the *n*=1 step occurs. For a frequency of *f*=2.7 GHz (red line), this first odd step is fully suppressed, showing an anomalous first step at *hf*/*e*. The presence or absence of the *n*=1 can be conveniently detected by binning the measurement data according to the voltage (with a 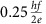 bin size). The resulting histograms of the voltage *V* are presented as bar plots in Fig. 2c. For *V*_*n*_=*nhf*/2*e* with *n* integer, Shapiro steps appear as peaks in the bin counts, the amplitude of which then reflects the length of the current step (in nA). For *f*=11.2 GHz, all steps emerge clearly from the background. For *f*=2.7 GHz, the peak at *V*=*hf*/2*e* is absent, reflecting the suppression of the *n*=1 Shapiro step. This anomalous behaviour of the Shapiro steps constitutes the main finding of this article. Below, we carefully analyse its origin and conclude that it indicates the existence of a 4*π*-periodic contribution to the supercurrent.

### Dependence on rf power

**Figure 3:**
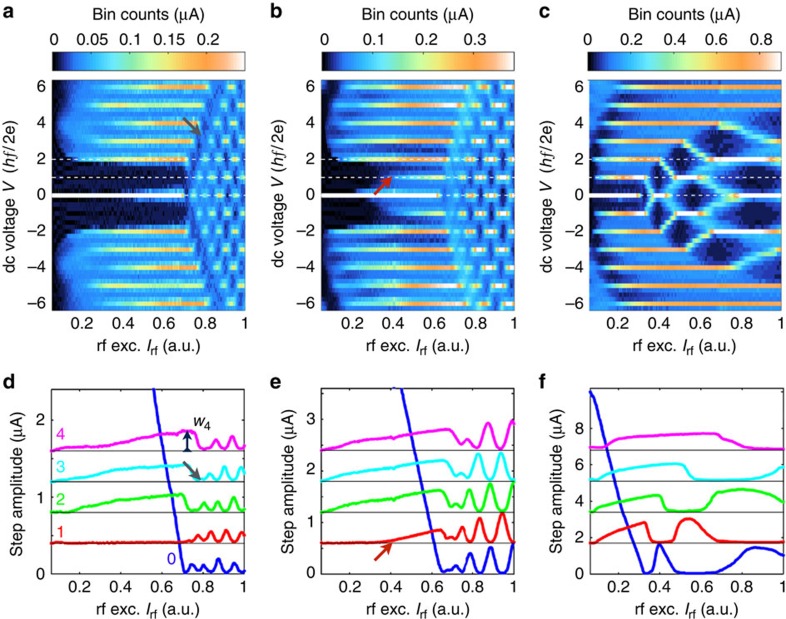
2D plots of the bin counts and Shapiro step amplitudes for the *L*=150-nm junction. (**a**–**c**) 2D map of the bin counts for frequencies *f*=2.7, 5.3 and 11.2 GHz, respectively. Shapiro steps are identified as maxima for constant voltages *V*_*n*_ (white dashed lines emphasize *n*=0, 1 and 2). For *f*=11.2 GHz, all steps are visible. When frequency is lowered (*f*=5.3 GHz), the first odd step (*n*=1) is absent up to a rf excitation indicated by the red arrow. Finally, at *f*=2.7 GHz, the first step is completely invisible up to the crossing point that marks the beginning of the oscillatory regime at high rf currents. A dark fringe (indicated by a dark grey arrow) is observed at finite voltages in the oscillating pattern concomitant with the missing *n*=1 step. (**d**–**f**) Horizontal linecuts through the previous colormaps (frequencies *f*=2.7, 5.3 and 11.2 GHz) that give access to the amplitudes of steps 0–4. While all Shapiro steps are clearly visible at high frequencies, the step *n*=1 progressively disappears as *f* decreases. From these plots, we access the maximum widths *w*_*n*_ of each step (see the example of *w*_4_ at *f*=2.7 GHz). For clarity, the different curves are offset by 0.4, 0.6 and 1.7 μA for **d**–**f**, respectively.

We now examine the crossover from high to low frequency, for which the first odd Shapiro step *n*=1 progressively disappears. To this end, we scan the presence of Shapiro steps for a range of rf powers at fixed frequencies and generate two-dimensional colour plots of the bin counts at the voltage *V* (which indicates the current height of the Shapiro step when present) as a function of the voltage *V* and rf current *I*_rf_. As shown in Fig. 3 (for the junction with *L*=150 nm), such plots reveal the presence of Shapiro steps as maxima at constant quantized voltages (horizontal lines). Let us first examine measurements taken at *f*=11.2 GHz. (Fig. 3c). At *I*_rf_=0, a single maximum at *V*=0 reflects the presence of a supercurrent. As *I*_rf_ increases, Shapiro steps progressively appear, starting from low values of *n*, while the amplitude of the supercurrent (*n*=0) decreases and eventually vanishes. At higher powers, the steps show an oscillatory pattern, reminiscent of Bessel functions occurring in the voltage bias case^33^,^34^. Horizontal linecuts at constant voltages give access to the amplitude of the first steps (*n*=0, 1, 2, 3 and 4), presented in the lower panels of Fig. 3 as a function of rf current *I*_rf_. For high frequencies such as *f*=11.2 GHz, our device exhibits the conventional behaviour that is seen in various other systems (carbon nanotubes^35^, graphene^36^ or Bi_2_*S*e_3_ (ref. 12) weak links), that always (regardless of frequency) show a clear presence of the *n*=1 step. The case of atomic contacts (with a few ballistic highly transparent modes) is particularly well understood, and also exhibits a strong *n*=1 Shapiro resonance in excellent agreement with theoretical models^37^,^38^. In the Supplementary Note 3 and Figs 11 and 12, we provide additional measurements on graphene-based devices (another example of two-dimensional Dirac material), that also show this standard behaviour in all accessible regimes.

In contrast to the conventional Shapiro features commonly reported, our HgTe-based junctions exhibit a very clear vanishing of the first step *n*=1 when the excitation frequency *f* is decreased. Measurements at *f*=5.3 GHz show that the first step is suppressed below a certain value of *I*_rf_ (indicated by the red arrow), and that it is completely absent at *f*=2.7 GHz. In the oscillatory regime at higher rf currents, a suppressed first oscillation (dark fringe indicated by the dark grey arrow) becomes clearly visible at low frequency, demonstrating the range of influence of the vanishing first step on the rest of the pattern. In the lower panels, a complete suppression of the first step or disturbances in the oscillations at higher rf currents can similarly be observed. This crossover has been observed on all working devices, up to 800 mK, which is the highest stable temperature accessible in our fridge. In some cases, hysteretic behaviour at low temperatures hinders the observation of low-index steps (Supplementary Fig. 8 and Note 2). However, importantly, biasing instabilities and sudden current switches (such as the ones observed in the hysteretic regime) can be excluded as a mechanism for the missing *n*=1 step, as the same features are seen in measurements of a junction in which bistability is suppressed by a shunt resistor (Supplementary Fig. 9 and Note 3).

In opposition to a missing *n*=1 step, additional subharmonic steps (for *n*=*p*/*q* fractional value) are often observed^37^,^39^ as a consequence of non-linearities, capacitance effects or higher harmonics in the CPR. Such higher harmonics have been predicted^5^ and detected^32^ in our junctions. At higher frequencies, we indeed observe half-integer steps (*n*=1/2, 3/2 and so on, Supplementary Note 3 and Fig. 6) but they clearly appear in a different regime from where we observe the missing *n*=1 step.

### Analysis and amplitude of the 4*π*-periodic supercurrent

**Figure 4:**
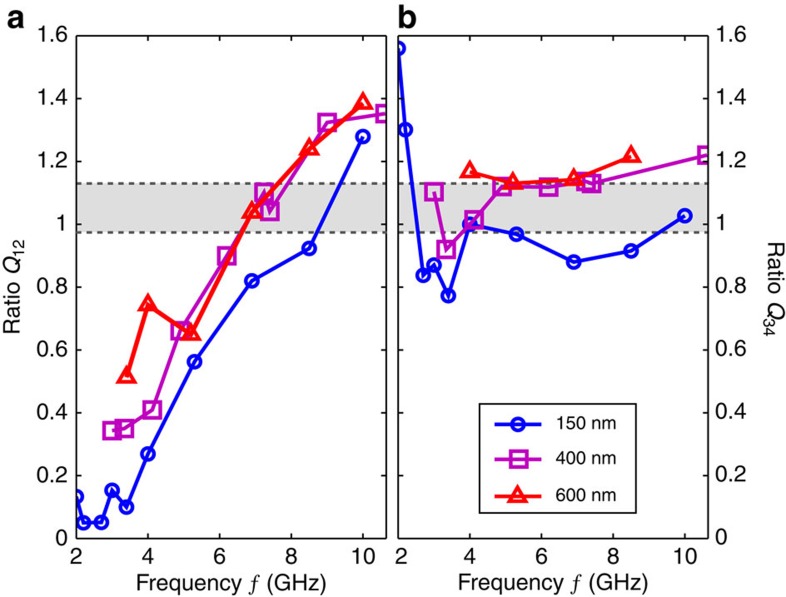
Ratios of step widths *Q*_12_ and *Q*_34_ versus frequency *f*. For each length *L* of the JJ, we calculate the ratios of step amplitudes *Q*_12_=*w*_1_/*w*_2_ (**a**) and *Q*_34_=*w*_3_/*w*_4_ (**b**) and plot them as a function of the rf frequency. *Q*_12_ shows a very clear decrease as frequency *f* is lowered. A minimum around 0.05 is obtained for the 150-nm junction, but we observe that this minimum tends to increase with the length *L* of the junction. In contrast, even if the measurements show some scattering, the ratio of higher order steps *Q*_34_ does not show significant variation. For comparisons, we evaluated *Q*_12_ and *Q*_34_ from a conventional RSJ model, and show the results as a grey area.

The presence of a 4*π*-periodic contribution in the supercurrent *I*_4*π*_ sin *φ*/2 is the only known mechanism to result in the observed doubling of the Shapiro step size. As already mentioned, microscopic models based on Bogoliubov-de Gennes equations have predicted such a 4*π*-periodic contribution in the CPR^2^,^3^,^4^,^5^, which originates from the presence of a gapless topological Andreev doublet. This anomalous CPR can then be supplemented with the Josephson equation on the time-evolution of the phase difference to simulate the dynamics of such a system (see Methods section). This dynamics is captured in the extended Resistively Shunted Junction (RSJ) model of Dominguez *et al.*^25^ It takes into account the presence of a sin *φ*/2 contribution in the supercurrent and explains the crossover between the two frequency regimes by the highly non-linear dynamics of the junction. When a small 4*π*-periodic contribution *I*_4*π*_ sin *φ*/2 is superposed on a large 2*π*-periodic supercurrent *I*_2*π*_ sin *φ* in the CPR, the latter dominates the high-frequency Shapiro response, but the weak 4*π*-periodic contribution is revealed at low frequencies by doubled Shapiro steps (Supplementary Note 4 and Figs 13, 14, 15). Doubled Shapiro steps are observed only when the driving frequency *f* becomes smaller than the characteristic frequency 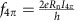 (with *R*_n_ the normal state resistance of the device). This frequency scale based on the amplitude of the 4*π*-periodic supercurrent is expected to be much smaller than the typical Josephson frequency scale 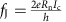 (*f*_J_≃53 GHz for the 150 nm long junction), as 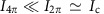. To estimate *I*_4*π*_, we introduce two indicators *Q*_12_ and *Q*_34_ as follows. From the maximum amplitude of the first lobe of each step, denoted by 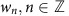, (see Fig. 3a where the measurement is indicated for the *n*=4 step), we define and compute the ratios *Q*_12_=*w*_1_/*w*_2_, *Q*_34_=*w*_3_/*w*_4_, and plot them as a function of the rf excitation frequency (Fig. 4). Despite some scattering, we observe a clear decrease of *Q*_12_ towards 0 with decreasing frequency, while *Q*_34_ remains constant around 1, for all lengths. For the shortest junction (150 nm) *Q*_12_ reaches a value of 0.05 around 2 GHz, and the first step *n*=1 is invisible. For comparison, we have also plotted the boundaries (grey dashed lines) between which the ratios *Q*_12_ and *Q*_34_ vary in the standard RSJ model^34^,^40^ (with only a sin *φ* component in the supercurrent). While the ratio *Q*_34_ remains close to the grey region, the behaviour of *Q*_12_ is not properly described. Assuming the validity of the above criterion, one can evaluate the number of 4*π*-periodic channels. We estimate *f*_4*π*_=4.5–5 GHz and *I*_4*π*_=250–300 nA for the 150-nm junction, and *f*_4*π*_=4 GHz and *I*_4*π*_=50–70 nA for the longer junctions (400 and 600 nm). One can compare these values with the maximum supercurrent carried by one channel^41^, given by *e*Δ_i_/*ħ* per channel where Δ_i_ can be estimated from the decay of *I*_c_ with temperature (Supplementary Figs 2 and 3 and Note 2). With Δ_i_=0.35 meV (150 nm) and Δ_i_=0.1–0.15 meV (400 and 600 nm), we estimate that the 4*π*-periodic contribution amounts to that of 1–3 channels that is compatible with the presence of one topological mode in our system, despite uncertainties on the exact value of *f*_4*π*_ and Δ_i_. Results on three other junctions with different parameters have been compiled (Supplementary Table 1) and are consistent with this estimate.

Finally, one might also suspect that the 4*π*-periodic contribution stems from Landau–Zener transitions occurring at the anticrossing (for *φ*=*π*[2*π*]), causing some highly transparent 2*π*-periodic states to behave effectively as 4*π*-periodic, in the absence of truly 4*π*-periodic modes. In a single mode model^25^, one can numerically show that the quantization of the Shapiro steps is lost when the Landau–Zener tunnelling probability is <1: the Shapiro steps split in two and depart from their quantized values *V*_*n*_, (*n* even), and eventually disappear for probabilities below 0.7. We do not experimentally observe such effects in any accessible regime. Assuming the validity of this specific model, an upper bound on the possible energy splitting 2*δ* between positive and negative energy branches can be evaluated from the Landau–Zener transition probability. Given our experimental resolution, we obtain the upper-bound *δ*≤9 μeV for the 400 and 600 nm junctions, and *δ*≤18 μeV for the 150 nm one (Supplementary Note 4 and Figs 16 and 17). This is much smaller than the energy scale given by the temperature (70 μeV at 800 mK) and corresponds in both cases to a transmission ≥0.994. Besides, there are no reports of missing odd Shapiro steps due to Landau–Zener transitions in highly ballistic junctions to date.

Interestingly, only the first step *n*=1 is missing (similarly to that reported for etched InSb nanowire devices^19^), and not the following odd steps *n*=3, 5 and so on. This feature is not well understood, but one can also speculate that the frequency *f* has to be lowered further for other odd steps to vanish, in a range that is not accessible in this experiment. An alternative explanation is given by enhanced relaxation (due, for example, to coupling to the continuum of states above the superconducting gap), with a characteristic time scale that decreases as voltage increases^15^,^42^. However, most models assume a voltage bias of the junction and a more detailed analysis of the current bias case is needed.

To conclude, we have presented robust evidence for a 4*π*-periodic contribution to the supercurrent flowing in JJs based on the 3D TI HgTe. The consistency of the measurements in Hall bars and JJs signals that our devices are well-controlled, with well-defined proximity-induced superconducting HgTe contacts connected via a ballistic HgTe surface. Under rf irradiation, a suppression of the first Shapiro step is observed at low frequencies and low magnetic fields, for a wide range of temperatures (up to 800 mK), which we attribute to the existence of a 4*π*-periodic component in the supercurrent. The study of its order of magnitude and of Landau–Zener transitions reveal that these experimental observations are compatible with the presence of a few 4*π*-periodic gapless Andreev bound states. The topologically non-trivial behaviour of HgTe has been established in the previous work, so that such states would likely stem from the topologically protected gapless Andreev doublet. However, our current observations cannot exclude the presence of trivial ballistic states. Further investigations are required to conclusively demonstrate the relationship of these observations to Majorana physics^4^,^43^. Besides, these observations with a 3D TI of strained HgTe are very encouraging for future experiments in which the weak link would consist of narrow HgTe quantum wells that exhibit the quantum spin Hall effect^44^, in which the total number of transport modes should be reduced to a few.

## Methods

### Sample preparation and layer characterization

Bulk HgTe layers are grown by molecular beam epitaxy on a CdTe substrate. Before the fabrication of the JJs, the transport properties of each layer are characterized by the measurement of longitudinal and transverse (Hall) resistance in a Hall bar geometry. From the longitudinal resistance at zero magnetic field, one can extract the mobility of the layer, while the density is obtained from a linear fit of the Hall resistance between 0 and 500 mT. The layers being very similar to the ones presented in refs 21, 22, we refer the interested reader to these references where the measurements are discussed in detail.

Using a Ti/SiO_2_ etch mask, the samples are patterned via Ar^+^ ion beam milling to obtain 2-μm wide HgTe stripes. The Nb contacts (60 nm Nb+Al/Ru cap) are sputtered on top of the HgTe layer, after a short Ar^+^-milling step to remove any adsorbant or reaction product left on the HgTe mesa after exposure to air. The top-layer of HgTe below the contact may be disordered due to the deposition of the Nb contacts. We assume that this top surface is proximitized by interaction with the Nb, but that the HgTe weak link between the contacts remains in the ballistic regime as highlighted in the main text.

In Supplementary Fig. 1, scanning electron microscopy pictures of a typical junction are shown. From these scanning electron microscopy pictures, it is observed that the nominal length (used in the article) probably overestimates the physical length of the JJs. We evaluate the physical length to be 60–70 nm smaller than the nominal length.

### Simulations using RSJ equations

The 4*π*-periodic contribution to the supercurrent has been theoretically investigated using microscopic models, in particular Bogoliubov-de Gennes hamiltonians^2^,^3^,^5^. However, difficulties arise when dynamics have to be taken into account. The RSJ model is to our knowledge the only way to define the time-averaged voltage measured in a current bias experiment like ours. The CPR derived from microscopic model can then be supplemented with the universally valid time-evolution of the phase difference of the Josephson effect, *dφ*/*dt*=2*eV*/*ħ*. In this framework, the junction is modelled together with a resistive shunt to capture the impedance of the environment (that plays an essential role in the dynamics of the junction). The total current through the system *I* can be written as the sum *I*=*I*_R_+*I*_*S*_ where 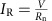 is the current through the resistor *R*_n_ and *I*_*S*_ the supercurrent through the junction. The geometrical capacitance is small, and we neglect it here^9^. Combining the first Josephson equation *dφ*/*dt*=2*eV*/*ħ*, and the current bias *I*=*I*_dc_+ *I*_rf_ sin 2*πft* obtains a first-order ordinary differential equation:


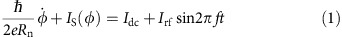


This model does not take into account all microscopic details (for example, the normal state resistance *R*_n_, which is assumed to be independent of voltage, which in reality will not be true). It has nonetheless the key aspects to capture the essential features of the dynamic Josephson current relevant to our observations. Simulations based on a standard RSJ model^34^ have been carried out to compare our results with a simple and well-understood model. The rf excitation is here represented as a current *I*_rf_ instead of a voltage *V*_rf_ in agreement with most of the literature on Shapiro steps. It assumes that the characteristic field impedance of the radiation field is high compared with the junction impedance^34^. In our case (low capacitance), the typical impedance of the junction is given by its resistance (typically between 30 and 150 Ω), smaller than the free space impedance (in which the rf excitation signal propagates before the junction) given by 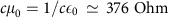. This approximation is probably a bit crude, but it is further justified by the overall agreement of the effect of frequency on the Shapiro response (Fig. 3 and Supplementary Figs 5, 13, 14.), which is a characteristic feature of the current bias model developed by Russer^34^. We simulate the results of this equation using a simple Runge-Kutta algorithm (RK4) to obtain the *I*–*V* curve, realize a binning of the voltage *V* and finally compute the ratios *Q*_12_, *Q*_34_ for comparison with experimental results.

## Additional information

**How to cite this article:** Wiedenmann, J. *et al.* 4*π*-periodic Josephson supercurrent in HgTe-based topological Josephson junctions. *Nat. Commun.* 7:10303 doi: 10.1038/ncomms10303 (2016).

## Supplementary Material

Supplementary Information Supplementary Figures 1-17, Supplementary Table 1, Supplementary Notes 1-4 and Supplementary References
