# Supplementary material for: 4π-periodic Josephson supercurrent in HgTe-based topological Josephson junctions
Source: Nat Commun. 2016 Jan 21;7:10303. doi: 10.1038/ncomms10303 (PMC4735757; doi:10.1038/ncomms10303)
Supplement: Supplementary Information — Supplementary Figures 1-17, Supplementary Table 1, Supplementary Notes 1-4 and Supplementary References [file ncomms10303-s1.pdf]

4π-periodic Josephson supercurrent

in HgTe-based topological Josephson junctions

– Supplementary Information –

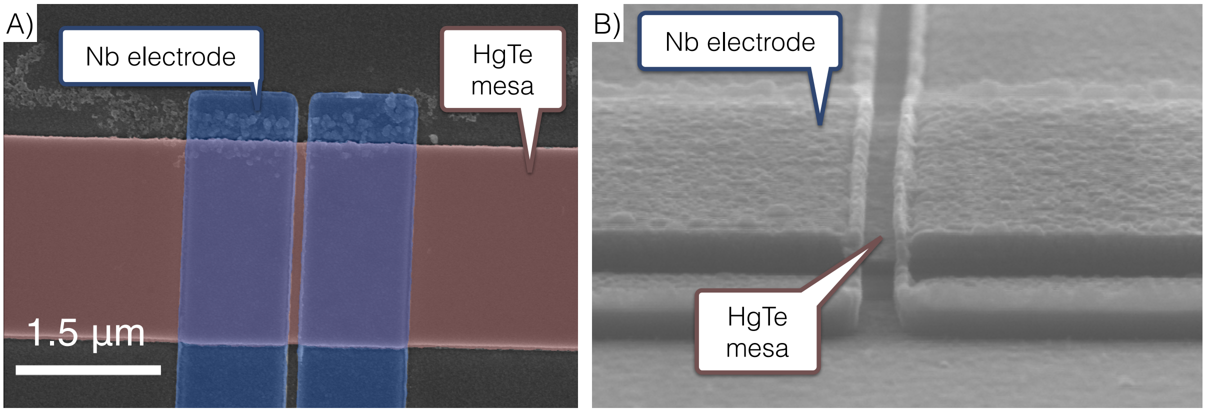

Supplementary Figure 1. **SEM pictures of two typical devices -** (A) Colorized SEM picture highlighting the HgTe mesa (in red) and the Nb electrodes (in blue), in the lateral junction geometry presented in Fig.1 (main text). (B) Close-up on the junction itself. Nb sidewalls are visible on the electrodes, and are a consequence of sputter deposition on the sidewalls of the resist pattern used to define contacts.

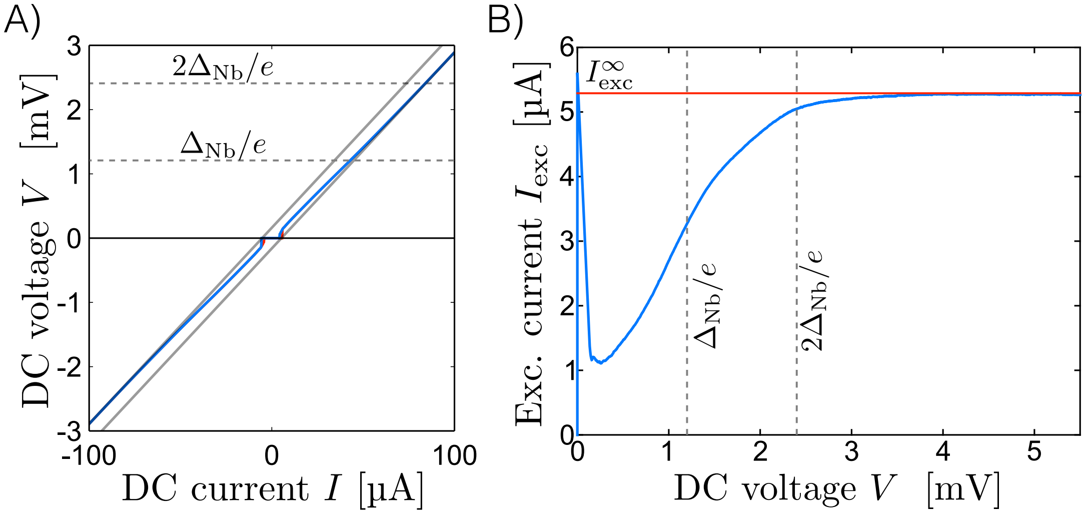

Supplementary Figure 2. **Excess current and midgap states -** (A) The I-V curve of the 150 nm junction is presented in blue and red line (for the two sweep directions). Two asymptotes are presented as a grey line, that do not cross the origin, demonstrating the presence of an excess current. Two horizontal dashed line have been added as guidelines and represent possible onsets at $eV \simeq \Delta_{\mathrm{Nb}}$ and $eV \simeq 2\Delta_{\mathrm{Nb}}$. (B) Excess current $I_{\mathrm{exc}}(V) = I - V/R_{\mathrm{n}}$ as a function of voltage V. Inflexions are visible around $V \simeq 1.4\,\mathrm{mV}$ and $V \simeq 2.4\,\mathrm{mV}$. At high bias, $I_{\mathrm{exc}}(V) \rightarrow I_{\mathrm{exc}}^{\infty} = 5.3\,\mu\mathrm{A}$.

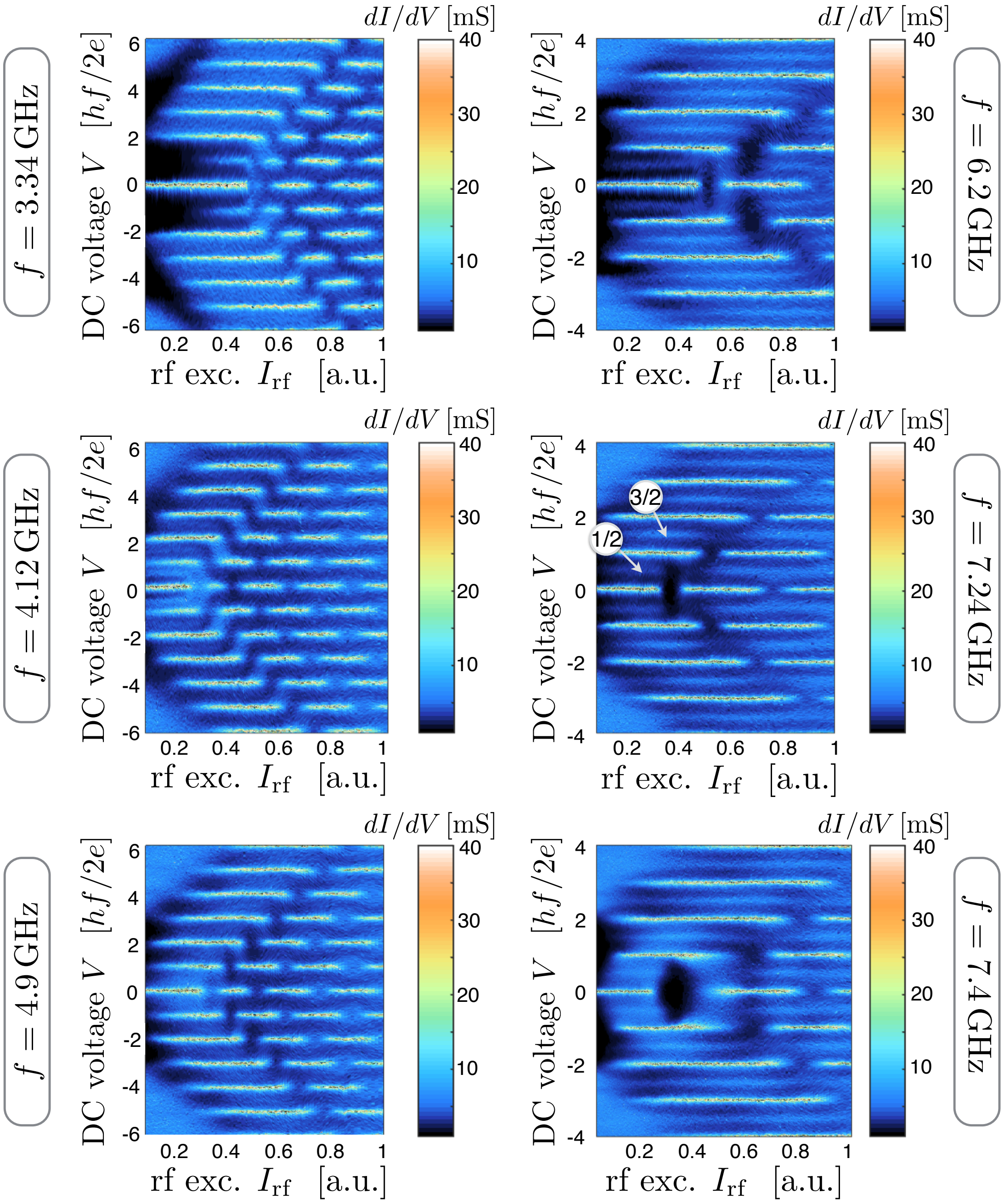

Supplementary Figure 3. **Frequency dependence of the Shapiro response -** The differential conductance dI/dV is plotted as a colorscale, as a function of the dc voltage V and rf current drive $I_{\rm rf}$ for different frequencies. The progressive appearance of the initially (partially) missing $n = 1$ step is observed. At high frequencies ($f \geq 6.2\,\text{GHz}$), subharmonic steps $n = 1/2, 3/2, 5/2...$ become visible.

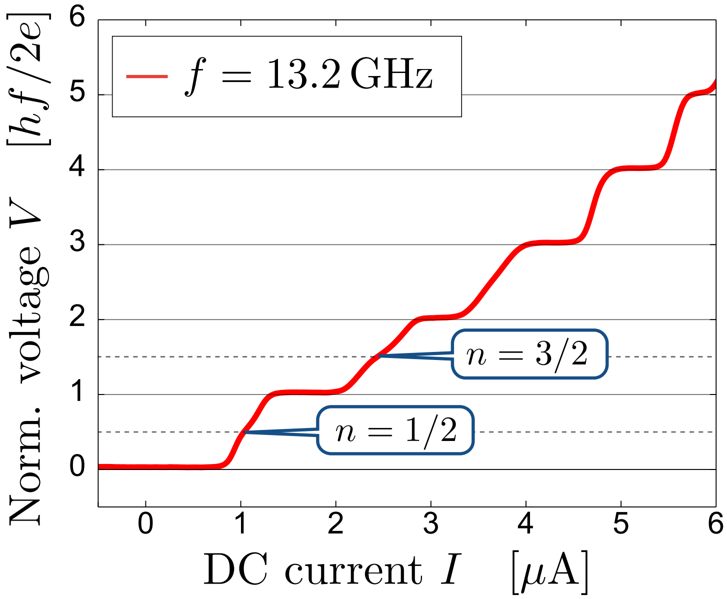

Supplementary Figure 4. **Subharmonic steps -** We present an I-V curve obtained under rf irradiation at $f = 13.2\,\mathrm{GHz}$. The subharmonic structure (clearly visible in the differential resistance dV/dI in Supplementary Fig.3) is related to the appearance of subharmonic steps with index $n = 1/2, 3/2....$ Here in particular, one observes the steps $n = 1/2$ and $n = 3/2$.

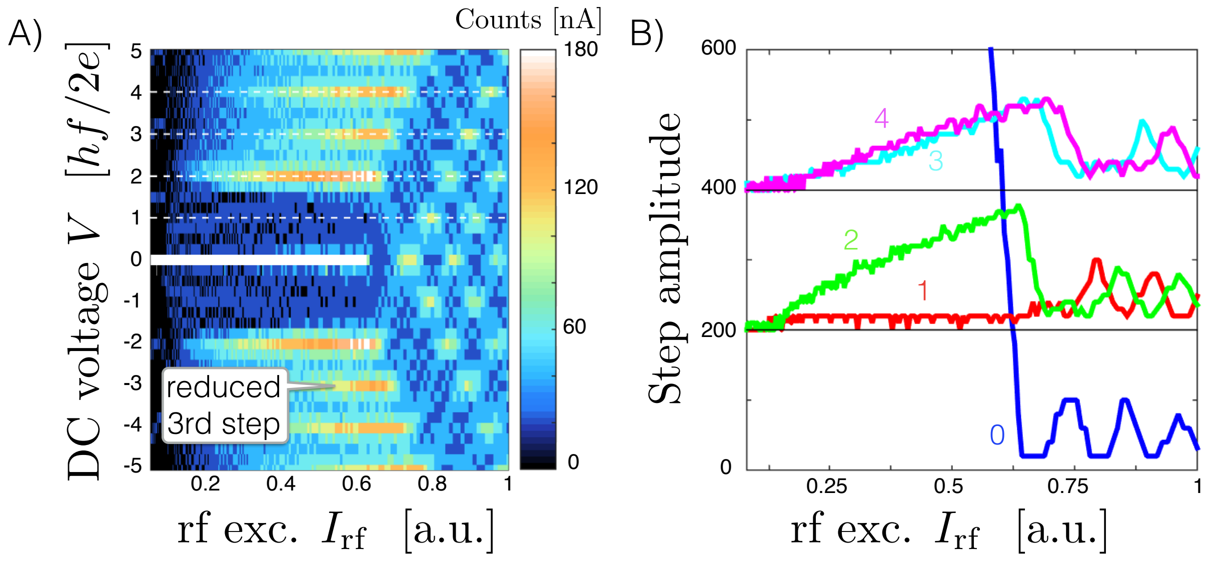

Supplementary Figure 5. **Weak reduction of the $n = 3$ step -** (A) The differential resistance dV/dI is plotted as a colorscale, as a function of the dc and rf current drives I and $I_{\rm rf}$ for a frequency of $f = 3\,$GHz. A weak suppression of the third step ($n = 3$) is observed for low rf currents. (B) The step amplitudes are extracted by binning voltages (see main text) and presented for steps 0 to 4 (with an offset of 200 nA for $n = 1, 2$ and 400 nA for $n = 3, 4$). As commonly observed in our samples, the first step (red line) if fully suppressed before the crossing point. More interestingly, the amplitude of the third step (cyan line) is reduced as compared with our usual observations. In particular, it remains smaller than the amplitude of the fourth step (presented in magenta)

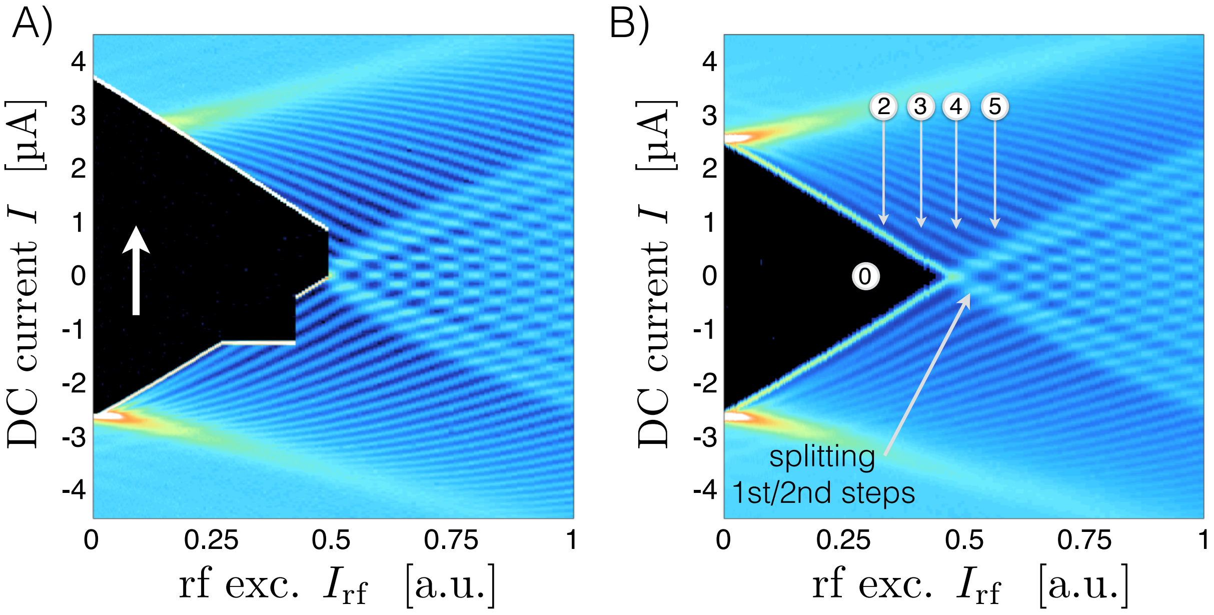

Supplementary Figure 6. **Effect of hysteresis on Shapiro steps -** The differential resistance dV/dI is plotted as a colorscale, as a function of the dc and rf current drives I and $I_{\rm rf}$ for a frequency of $f = 3\,\mathrm{GHz}$, at the base temperature of the dilution refrigerator $T = 12\,\mathrm{mK}$ (A) and at $T = 800\,\mathrm{mK}$ (B). The asymmetry in the figure signals a strong hysteretic behavior in the I-V curve (see main text, Fig.2). The wide white arrow (A) symbolizes the sweep direction of the dc bias current I. In the hysteretic region, low index n Shapiro steps are not visible. However, for high rf power, hysteresis vanishes and Shapiro steps reappear. The typical splitting of the $n = 1/n = 2$ steps is in particular still observable. In contrast, all steps (apart from the missing $n = 1$) are visible at $T = 800\,\mathrm{mK}$, and no asymmetry is observed.

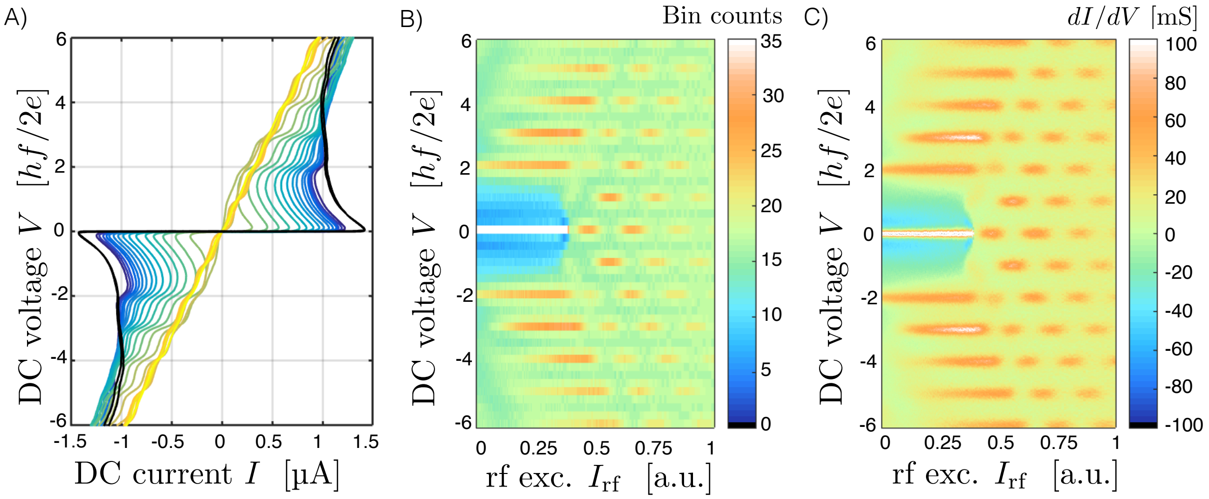

Supplementary Figure 7. **Shapiro steps on a shunted device -** (A) $I{-}V$ curves in the shunted configuration, at temperature $T = 12\,\mathrm{mK}$. A measurement in the absence of rf irradiation is shown as a black line. The low voltage regime with $dV/dI < 0$ corresponds to an unstable region when a pure current bias is applied. The colored lines present data measured under irradiation at $f = 4.2\,\mathrm{GHz}$. The rf current is increased progressively from indigo to yellow lines. (B) and (C) Histogram of the voltage and differential conductance dI/dV as a function of DC voltage V and rf current I_{rf}. As shown in the standard current bias configuration, a clear vanishing of the $n = 1$ Shapiro step is observed.

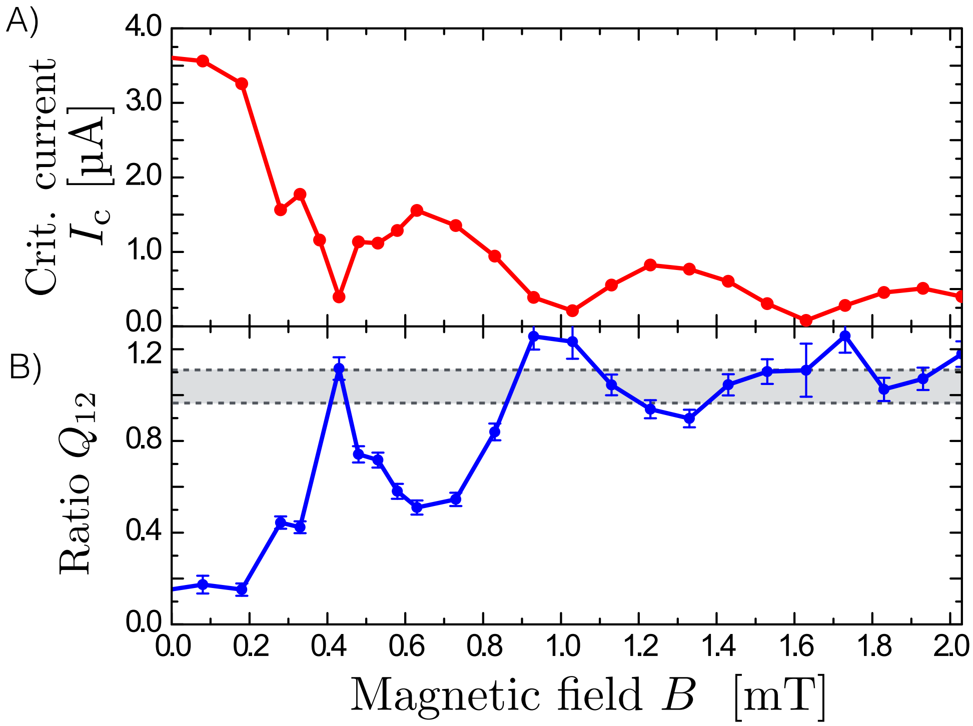

Supplementary Figure 8. **Magnetic field dependence -** (A) Critical current I_c as a function of the perpendicular magnetic field B. The typical diffraction pattern of the supercurrent is observed. (B) The ratio Q_{12} is presented as a blue line, as a function of the perpendicular magnetic field B. It follows the same oscillations as the critical current I_c. The grey area between the dashed lines show the region in which the ratio Q_{12} falls, as evaluated by a RSJ model.

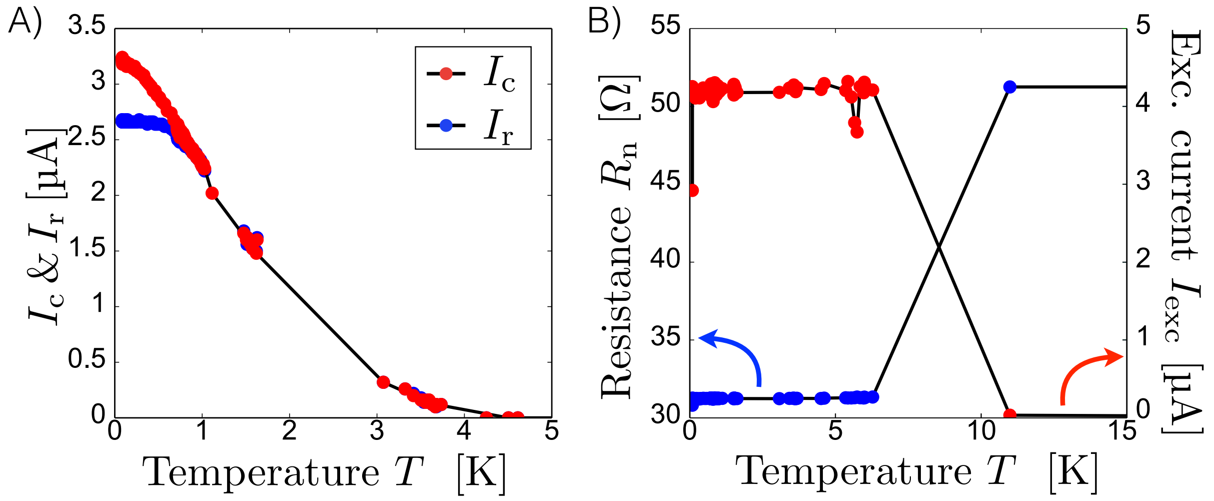

Supplementary Figure 9. **Temperature dependence -** (A) Temperature dependence of the critical and retrapping current I_c and I_c. The hysteretic region is seen for $T < 800\,\mathrm{mK}$ typically, for which $I_c < I_c$. (B) Temperature dependence of the excess current I_{exc} and normal state resistance R_n. The superconducting phase transition in the Nb contacts is seen here as a shift in the resistance R_n, and typically takes place at $T_c \simeq 8\,\mathrm{K}$. Simultaneously, the excess current I_{exc} exhibits a jump from zero to a finite value, thus reflecting the presence of Andreev reflections at the interfaces of the junction for temperatures $T < T_c$.

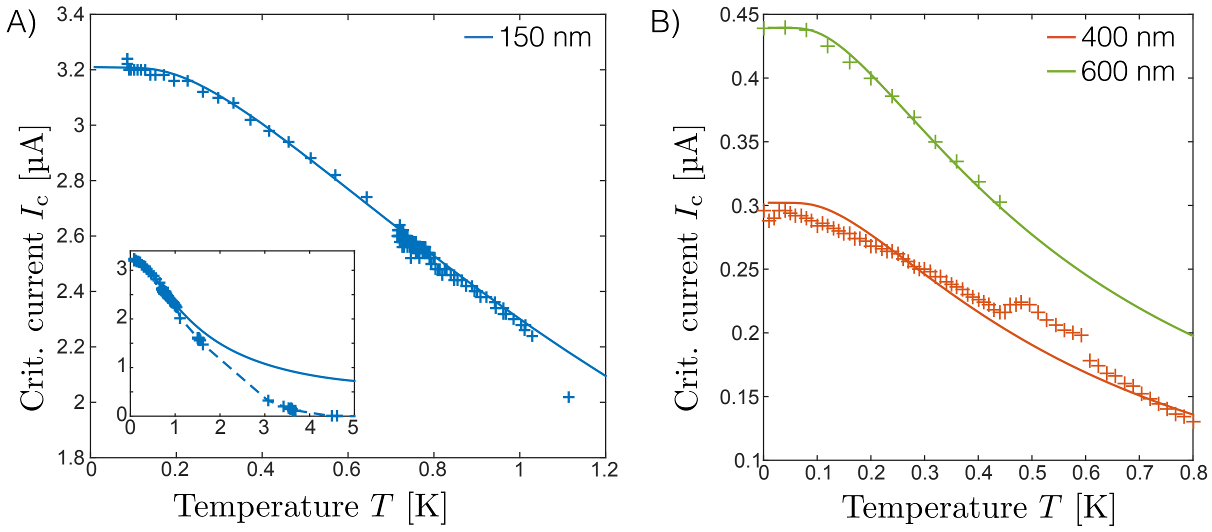

Supplementary Figure 10. **Fit of the critical current vs. temperature -** (A) Experimental data for the critical current $I_c(T)$ as a function of temperature T is presented as blue + symbols, for the 150 nm JJ. The solid line is obtained using Supplementary Eq.(1) for three-dimensional topological Josephson junctions, with $\Delta_i = 0.35\,\text{meV}$, $N = 117$, $Z = 0.1$. (inset) Complete temperature range. Beyond 1K, data is taken as the fridge warms up, resulting in inaccurate measurements of T. The fit departs from experimental data as the perturbative model breaks down. (B) The temperature dependence of $I_c(T)$ is presented for the 400 and 600 nm junctions, in red and green respectively. Experimental data is showed as + symbols while solid lines present fits with parameters $\Delta_i = 0.13\,\text{meV}$, $N = 48$, $Z = 1$ (600 nm) and $\Delta_i = 0.13\,\text{meV}$, $N = 33$, $Z = 1$ (400 nm).

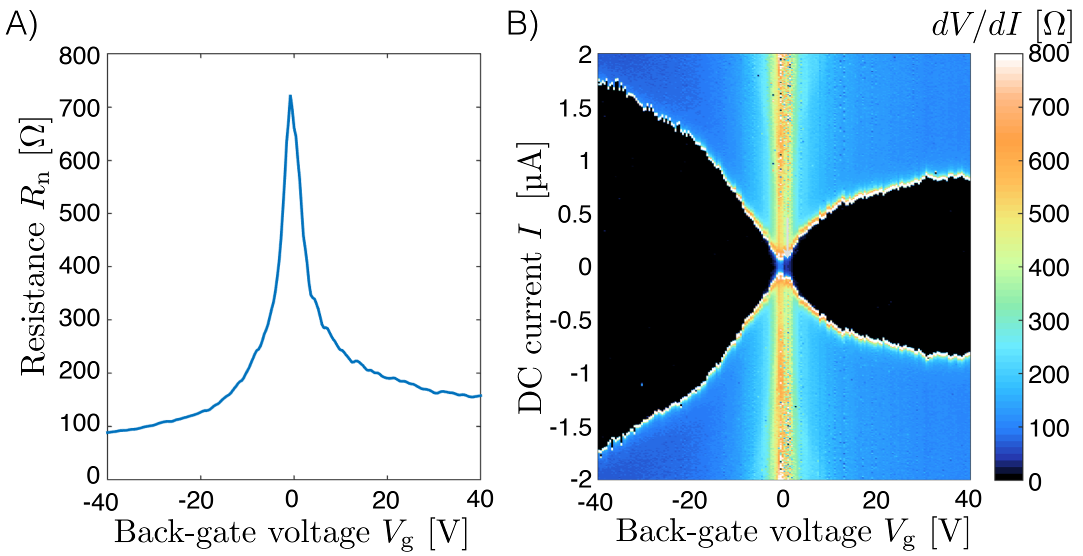

Supplementary Figure 11. **DC characterization of Josephson junctions with graphene weak link -** (A) Normal state resistance R_n of a graphene-based junction as a function of back-voltage V_g. The Dirac point is clearly indicated by a maximum around $V_g \simeq -1V$. (B) Colormap of the differential resistance dV/dI as a function of the back-gate voltage V_g and dc drive current I. The supercurrent is visible as a black region ($dV/dI \simeq 0$) over the whole back-gate range, ranging from a few tens of nA around the Dirac point up to 1.8 μA for large gate voltages.

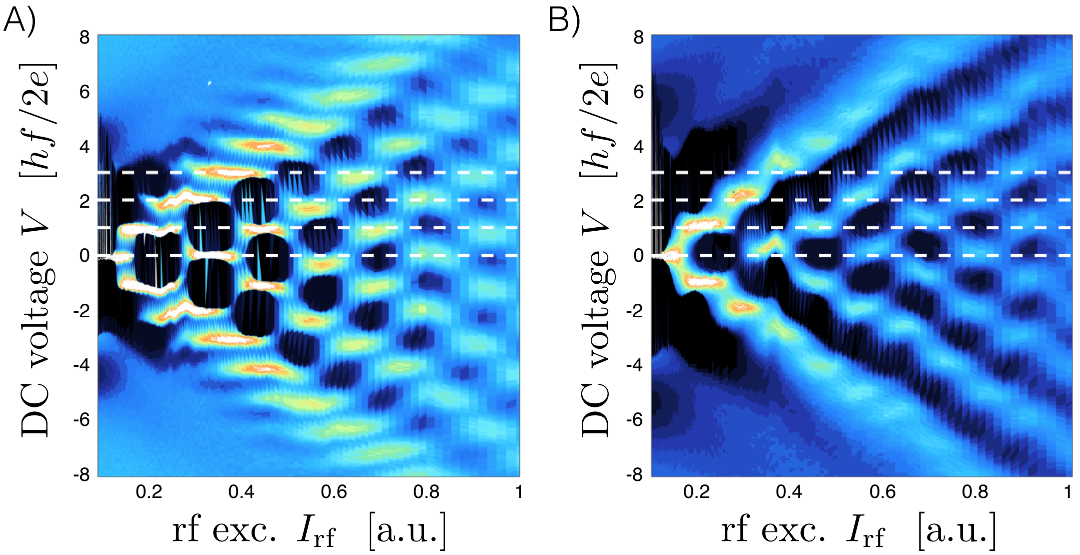

Supplementary Figure 12. **Shapiro response in graphene-based junctions -** The differential conductance dI/dV is plotted as a colorscale, as a function of the dc voltage V and rf current drives $I_{\rm rf}$ for two different frequencies, $f = 7\,\mathrm{GHz}$ (A) and $5.5\,\mathrm{GHz}$ (B). The data has been taken at the base temperature of the dilution refrigerator ($T = 25\,\mathrm{mK}$), close to the Dirac point. All Shapiro steps are visible, up to $|n| > 12$.

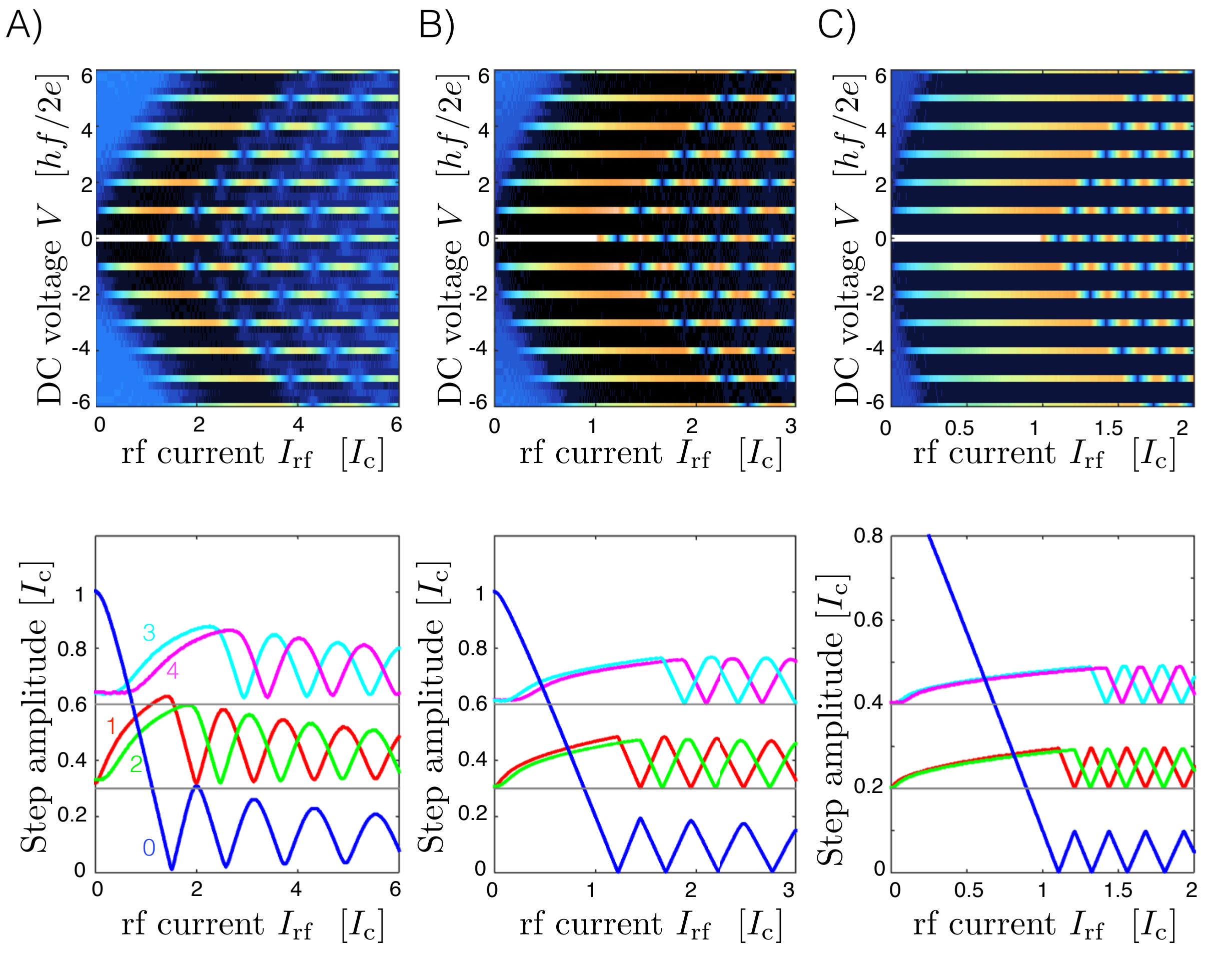

Supplementary Figure 13. **Simulated Shapiro response of a conventional Josephson junction -** In the framework of the RSJ model, we obtain colormaps of the bin counts as a function of the voltage bins (in normalized units) and rf current drive $I_{\rm rf}$. For a high frequency ($f = 0.4 f_{\rm J}$,A) the results obtained resembles that of Supplementary Fig.3C in the main text. All steps are clearly visible and appear one by one as $I_{\rm rf}$ increases. As frequency f is decreased ($f = 0.2 f_{\rm J}$ for B, $f = 0.1 f_{\rm J}$ for C) the pinch-off of the supercurrent is moved to higher powers. One also observes a decreasing period in the oscillating pattern.

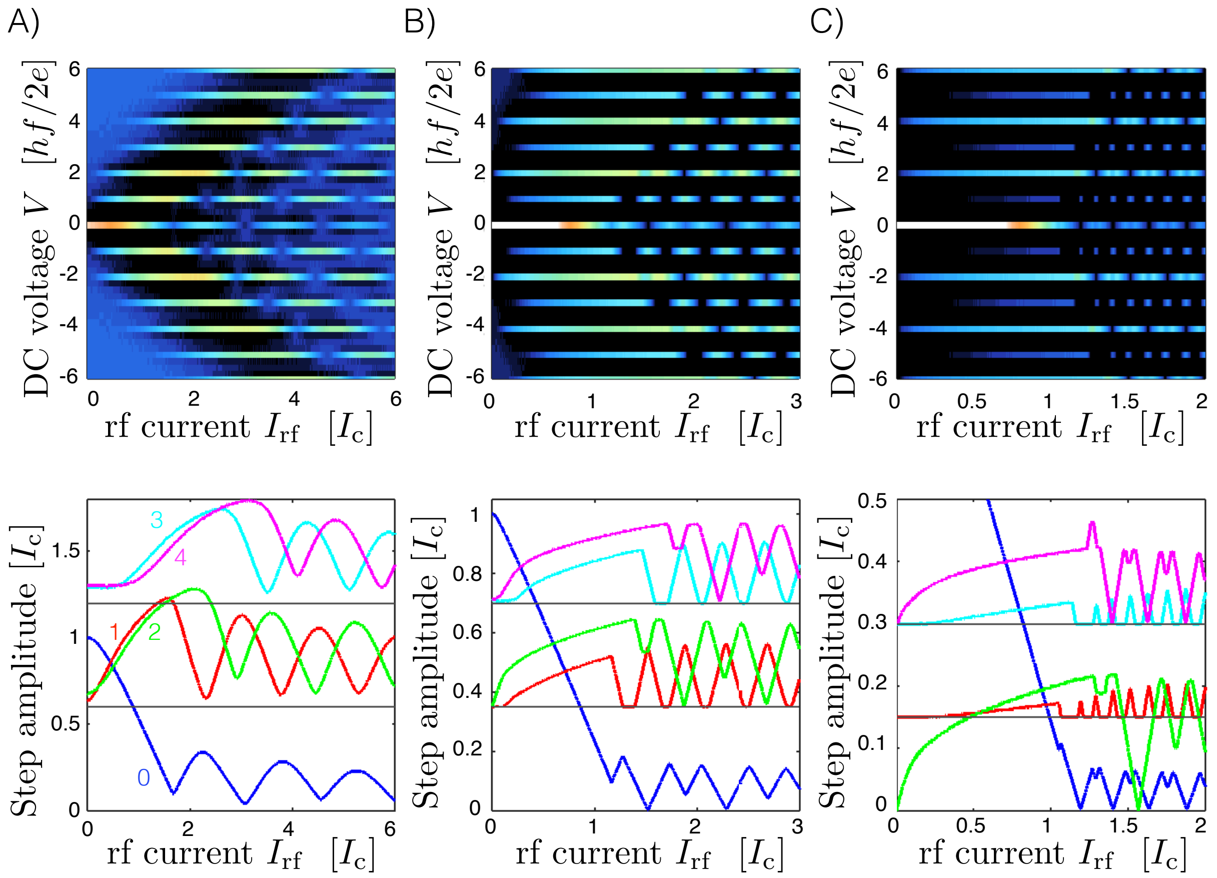

Supplementary Figure 14. **Simulated Shapiro response with a 4π-periodic term -** Colormaps of the bin counts as a function of the voltage (in normalized units) and rf current drive $I_{\rm rf}$, in the presence of a small 4π-periodic contribution (such that $f_{4\pi} = 0.15 f_{\rm J}$), and extracted amplitudes of step $n = 0$ to $n = 4$, as a function of the rf current drive $I_{\rm rf}$. At high frequency ($f = 0.5 f_{\rm J} > f_{4\pi}$ for A), all steps are visible, and the result is very similar to the one obtained without 4π-periodic modes. As the frequency is decreased ($f = f_{4\pi} = 0.15 f_{\rm J}$ for B and $f = 0.05 f_{\rm J}$ for C), the amplitude of the odd steps decreases, in particular the $n = 1$ step which is completely suppressed at low power. Simultaneously, the oscillatory pattern (high power) develops anomalies, with very pronounced first minima on the odd steps.

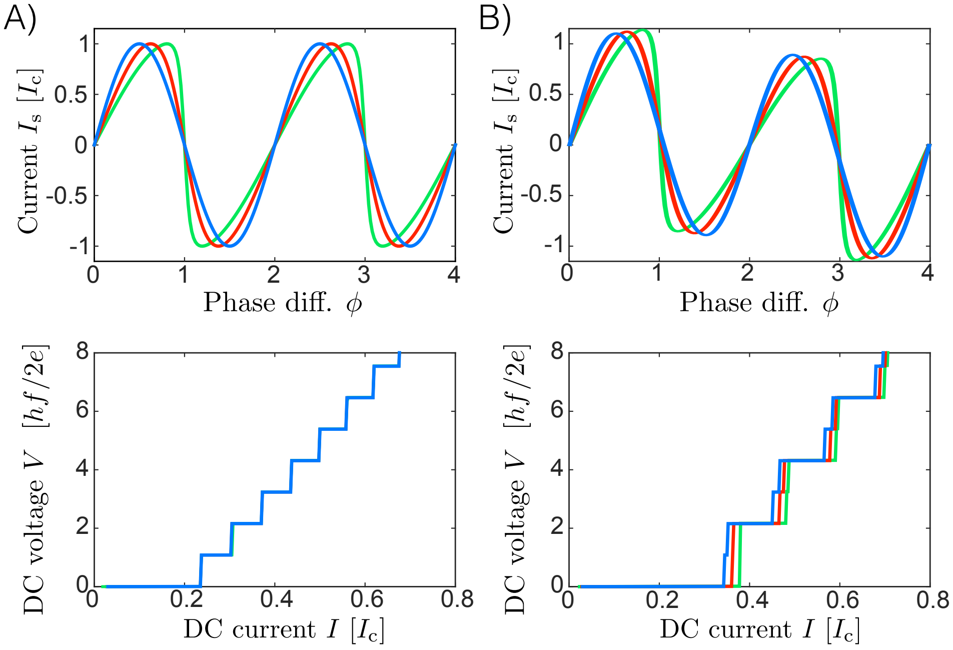

Supplementary Figure 15. **Effect of the CPR -** Investigated current-phase relations and simulated I-V curves in the presence of rf excitation. As a blue line, the sinusoidal 2-periodic supercurrent $I_{2\pi} \sin\phi$, in red and green, single-mode-like supercurrent with transmission $\tau = 0.8$ and 0.99 respectively (A). In panel (B), an additional 4π-periodic contribution is added, with amplitude $I_{4\pi} = 0.15 I_{2\pi}$. While all steps are visible in the I-V curve in the absence of 4-periodic modes, odd steps are missing or strongly suppressed when they are present. The exact CPR plays a small role in the crossover from the 4π-dominated regime to the conventional one when frequency is increased.

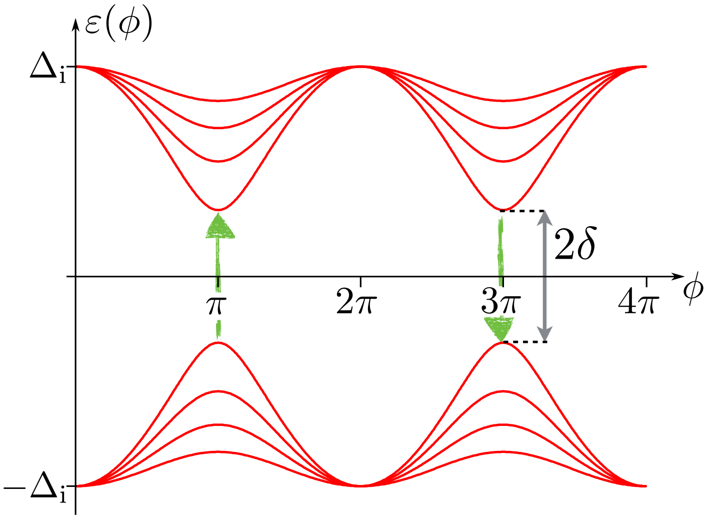

Supplementary Figure 16. **Effect of Landau-Zener transitions -** A typical spectrum of gapped 2π-periodic Andreev levels is presented as a function of the superconducting phase difference ϕ. In the presence of Landau-Zener transitions at the anticrossings (represented as green arrows for $\phi = \pi, 3\pi, ...$), some energy levels with small gap δ (high transmission) could contribute as a 4π-periodic component in the supercurrent.

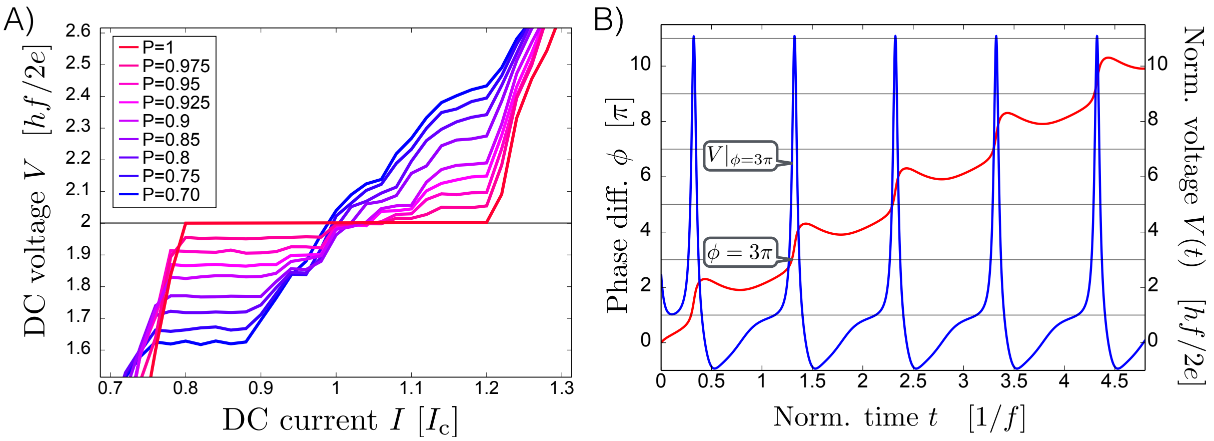

Supplementary Figure 17. **Landau-Zener transitions in the RSJ model -** (A) Evolution of the Shapiro step $n = 2$: I-V curves obtained for different values of the Landau-Zener probability P are plotted, with a focus on the $n = 2$ Shapiro step. When $P = 1$, the second step ($n = 2$) is fully developed and reaches the expected quantized value hf/e. For $P < 1$, it progressively deteriorates: the voltage departs from the quantized value $hf/2e$, and the plateau becomes less visible. (B) Time evolution of phase difference $\phi(t)$ and voltage $V(t)$: The phase difference $\phi(t)$ (red line, in units of π) and voltage $V(t)$ (blue line, in units $hf/2e$) across the junction is calculated via RSJ equations and plotted as a function of time t in units of the rf period $1/f$. The estimation of the voltage $V|_{3\pi}$ at $\phi(t) = 3\pi$ (anticrossing) enables a proper evaluation of the Landau-Zener transition probability.

	L (nm)	I_c (µA)	R_n (Ω)	f_J (GHz)	$f_{4\pi}$ (GHz)	$I_{4\pi}$ (nA)	Δ_i (meV)	i_0 (nA)	$N_{4\pi}$
A	150	$3.3^{\dagger}$	33	53	4.5-5	250-300	0.35	90	3
B	400	0.29	158	22	4	55	0.1-0.15	25-40	2
C	600	0.44	165	35	4	50	0.1-0.15	25-40	1-2
D	150	1.5	82	59	3-4	75-100	$0.2\text{-}0.25^{*}$	50-65	1-2
E	200	4.4	52	110	4-5	160-200	$0.4\text{-}0.5^{*}$	100-130	1-2
F	200	5.2	56	138	4-5	150-190	$0.4\text{-}0.5^{*}$	100-130	1-2

Supplementary Table I. **Summary of measured and estimated parameters -** In this table, we present the parameters measured or estimated on 6 different devices: length L, critical current I_c, normal state resistance R_n, Josephson characteristic frequency f_J, crossover frequency $f_{4\pi}$, 4π-periodic supercurrent, induced gap Δ_i, maximum current per channel i_0, number of 4π-periodic modes.

SUPPLEMENTARY NOTE 1 : ADDITIONAL CHARACTERIZATION MEASUREMENTS

A. Excess current

The excess current in our data is indeed strikingly present in all our samples. We take it as an additional indication of the systematic reproducibility of our data within a large variety of samples of different fabrication runs and measured in different cryostats at different locations. The excess current in a Josephson junction is obtained for voltages beyond 2Δ, as discussed by Blonder *et al.*[2], and reaches in principle twice the value for a NS contact. However, the amplitude of the excess current depends on elastic scattering at the interfaces and as such is not a sufficient measure. The presence of gapless modes can in principle be detected by a halved onset on the excess current: the asymptotic regime is reached for a bias eV on the order of the gap Δ and not 2Δ in the conventional case.

To analyze more precisely this behavior, we plot in the right panel of Supplementary Fig.2 the difference $I_{\rm exc}(V) = I - V/R_{\rm n}$, with $I_{\rm exc}(V) \to I_{\rm exc}^{\infty}$ for $eV \gg 2\Delta_{\rm Nb}$. We observe two inflexions around $V \simeq 1.4\,{\rm mV}$ and $V \simeq 2.4\,{\rm mV}$, that could be related to $\Delta_{\rm Nb}$ and $2\Delta_{\rm Nb}$. However, these features are relatively weak, and no clear transition at $eV \simeq \Delta_{\rm Nb}$ is observed.

The fact that we cannot observe a clear asymptotic regime at Δ is however not unexpected as the previous model is only directly applicable to N-S contacts, in which a single superconducting contact is involved and thus the dynamics of Josephson effect is not present. In a Josephson S-S contact, the observed currents and voltages below 2Δ are mixed with the time-averaged quantities of the Josephson effect. The I-V curve thus reveals a time-averaged part due to the Josephson effect and a static part due to the Andreev reflection processes. To get experimental access to the excess current part, it is necessary to separate these contributions. The contributions due to the Josephson effect are strongest at $V = 0$ and at finite voltages close to $V = 0$ but gradually decay in amplitude with increasing voltage. In contrast the excess current is present out to

high voltages beyond 2Δ and perhaps beyond Δ. Careful experimental work is therefore needed to disentangle these two processes from the experimental data. Furthermore, the theory has been developed for nanowires (single-band and short junction limit) with a simple representation of induced superconductivity, by a unique gap Δ. The fact that our system is a 2D surface state rather than a nanowire is expected to have little consequence. However, we expect to have a very different density of states due to the presence of two superconducting gaps in proximity with each other (the bulk Nb gap Δ_{Nb} and the induced gap Δ_{i}). The literature[5,6] shows for example the presence of features at both Δ_{Nb} and Δ_{i}, as well as different densities of states in regions of energy $\Delta_{\mathrm{i}} < \epsilon < \Delta_{\mathrm{Nb}}$ and $\epsilon > \Delta_{\mathrm{Nb}}$. The onset of the excess current in our measurements is of the same order of magnitude as $\Delta_{\mathrm{Nb}} \simeq 1\,\mathrm{meV}$, and the role of the induced gap (evaluated around $\Delta_{\mathrm{i}} \simeq 0.1 - 0.4\,\mathrm{meV}$) remains experimentally not accessible.

B. Temperature dependence

We present in Supplementary Fig.9 the temperature dependence of four quantities. In Supplementary Fig.9A, the critical current I_{c} and retrapping current I_{c} are shown (in red and blue dots respectively). Both decay on a typical scale of 1-2 K. The hysteretic region, characterized by $I_{\mathrm{c}} > I_{\mathrm{r}}$ takes place at roughly $T \leq 800\,\mathrm{mK}$ in most samples. Beyond this temperature, $I_{\mathrm{c}} = I_{\mathrm{r}}$ and no hysteresis is observed in the $I - V$ curve. In Supplementary Fig.9B, the excess current I_{exc} and the normal state resistance R_{n} of the junction are plotted as a function of temperature T. First, the resistance R_{n} (red dots) exhibits a jump (from $31\,\Omega$ to $51\,\Omega$) indicating the superconducting transition of the bulk Nb contacts. These measurements were taken in a dilution fridge for which stable temperature control was only possible below 1 K. Higher temperature measurements were collected while the system warmed up allowing only a few reliable measurement points such that the exact transition temperature is not known. From other measurements during cool down, we obtain a critical temperature around $T_{\mathrm{c}} \simeq 8\,\mathrm{K}$, slightly smaller than that of pure high-quality Nb (9.2 K) and compatible with the measurements presented

here. Simultaneously, the excess current is measured and exhibits a jump from zero to a finite value (around 4 µA) as the Nb contacts become superconducting. This signals the presence of Andreev reflections at both interfaces of the junctions for all temperatures $T < T_c$.

The temperature dependence of the junction with $L = 150$ nm presented in Supplementary Fig.9 can be analyzed using the approach developed by Tkachov *et al.*[1]. Each mode (indexed by the transverse component of the wavevector k_y) is described by an angle-dependent transmission T_θ where $\sin\theta = k_y/k_F$. The distribution of transmission T_θ reflects Klein tunneling through a barrier (characterized by a parameter Z in a BTK-like approach[2]), with topological protection of the mode $k_y = 0$ ($T_{\theta=0} = 1$). This model also takes into account induced superconductivity in the HgTe reservoir using a McMillan tunneling approach[3], which allows for the evaluation of the induced gap Δ_i. Unfortunately, this perturbative approach breaks down for high temperature when the induced gap Δ_i becomes comparable to the Nb gap Δ_{Nb} (that decreases with temperature).

$$I_c(T) = \frac{e\Gamma}{4\hbar}\sum_{k_y} \sin\phi\cos\theta \frac{T_\theta(1-\gamma+\frac{5}{2}\gamma^2-\frac{3}{2}\gamma^2 T_\theta \sin^2\frac{\phi}{2})}{1-T_\theta\sin^2\frac{\phi}{2}}$$
$$\tanh\Gamma\frac{(1-\gamma+\gamma^2)(1-T_\theta\sin^2\frac{\phi}{2})^{1/2}+\frac{1}{2}\gamma^2(1-T_\theta\sin^2\frac{\phi}{2})^{3/2}}{2k_BT} \tag{1}$$
$$T_\theta = \frac{\cos^2\theta}{1-\sin^2\theta/(1+Z^2)},\ \gamma = \Gamma/\Delta_{Nb},\ \Delta_i = \Gamma(1-\gamma+\frac{3}{2}\gamma^2)$$

Supplementary Eq.(1) has three fit parameters: the tunneling strength Γ (or equivalently the induced gap Δ_i), the barrier parameter Z and the number of modes N. As seen on Supplementary Fig.10A, the agreement below 1 K is very good for the $L = 150$ nm junction, but the fit diverges rapidly at high temperature. We obtain the following fitting parameters $\Gamma = 0.4$ meV, $\Delta_i = 0.35$ meV, $Z = 0.1, N = 117$. The number of modes is then in agreement with independent estimates from Hall bar measurements, and yields an estimate of Δ_i that can be used to estimate the number of 4π-periodic modes (see Supplementary Table 1).

For longer junctions ($L = 400 - 600$ nm), the agreement is not as good as for the 150 nm long junction presented here. The evaluation of the temperature scale over which

the decay of I_c is observed gives an estimate of $\Delta_i = 0.1 - 0.15\,\mathrm{meV}$ lower than for the junction with $L = 150\,\mathrm{nm}$. The amplitude of the critical current I_c decreases rapidly with length, and yields a number of modes $N < 50$ which seems unreasonably small given the estimate of the density from a separate Hall-bar, or from the value of N in the 150 nm long device on the same sample. It could indicate that these junctions depart from the short junction limit $l \ll \xi$ where ξ is the coherence length in the system. The natural coherence length $\xi_0 = \frac{\hbar v_F}{\pi \Delta_i}$ is typically between 250 and 1000 nm in our system[4]. For systems with mean free path $l \sim \xi_0$, the relevant length is in fact $\xi = \sqrt{\xi_0 l}$ in the range of 250 to 550 nm which is compatible with our findings.

SUPPLEMENTARY NOTE 2 : ADDITIONAL RESULTS ON HgTe-BASED JUNCTIONS

A. Frequency dependence and half-integer steps

In Supplementary Fig.3, we present datasets measured for a different sample than the one presented in the main text with length $L = 400\,\mathrm{nm}$. In particular, we show the transition from a doubled Shapiro step (at $f = 3.34\,\mathrm{GHz}$) to a regime in which the first step is fully recovered. At high frequencies (for $f \geq 6.2\,\mathrm{GHz}$), we observe the appearance of new half-integer steps, at voltages given by the step indexes $n = 1/2, 3/2, 5/2, ...$ To visulaize more clearly the subharmonic steps, we introduce a different way of visualizing our data. The differential conductance dI/dV is plotted as a colorscale, as a function of the dc voltage V and rf current drive I_{rf}. Thus, Shapiro steps appear as maxima (dI/dV diverges) for constant voltages, similarly to what is seen in the bin counts presented in the main text. In such plots, the information on the step amplitude is lost, but subharmonic steps of small amplitudes become more visible.

At $f = 3.34\,\mathrm{GHz}$, the first step ($n = 1$) is partially suppressed, but is recovered as frequency increases. The change in the oscillatory pattern is also clearly visible: at low frequency, fast oscillations follow a long region in which step $n = 0$ (supercurrent) is

present. As f is increased, oscillations start earlier and with a larger period. These features are accounted for by the RSJ model presented in section **??**. More interestingly, half-integer subharmonic steps ($n = 1/2, 3/2, 5/2...$) become clearly visible on the right panels (for $f \geq 6.2\,\mathrm{GHz}$).

As discussed in ref.7, it can be reproduced from the RSJ equations by adding a capacitive shunt in the circuit (RCSJ model[8]). Another possible mechanism is given by the presence of higher order harmonics in the current-phase relation. As a $\sin\phi/2$ term can suppress the odd steps, a $\sin 2\phi$ term generates subharmonic steps. While the geometric capacitance is believed to be negligible (see estimate in ref.9), a non-sinusoidal current phase relation has clearly been established recently in our junctions[4].

For the sake of completeness, we present in Supplementary Fig.4 a dataset measured at $f = 13.2\,\mathrm{GHz}$, on the junction with a length $L = 150\,\mathrm{nm}$ presented in main text. The I-V curve establishes the presence of two weak half-integer steps at $n = 1/2$ and $3/2$.

B. Summary of measured devices

In Supplementary Table 1, we summarize the parameters of 6 devices for which a complete set of data has been collected. Parameters are as follows : length L, critical current I_c, normal state resistance R_n, Josephson frequency $f_\mathrm{J} = \frac{2eR_\mathrm{n}I_\mathrm{c}}{h}$, crossover frequency $f_{4\pi}$, 4π-periodic contribution to the supercurrent $I_{4\pi}$, induced gap Δ_i, maximum current per mode $i_0 = \frac{e\Delta_\mathrm{i}}{h}$, and finally number of 4π-periodic modes. Starred (*) values indicate devices for which the temperature dependence of the critical current has not been measured precisely, so that Δ_i is only estimated from the value of I_c at base temperature, with large error bars. Despite a wide range of parameters explored, all junctions yield an estimated number of 4π-periodic modes between 1 and 3. † indicates the critical current of the junction with $L = 150\,\mathrm{nm}$ presented in the main text at the time when the Shapiro steps have been measured. A previous cool-down of the sample yielded $I_\mathrm{c} = 5.6\,\mathrm{\mu A}$ as presented in the main text (Fig.2). Aging of the sample has been observed in several samples and could explain this discrepancy.

C. Weak reduction of the $n = 3$ step

In Supplementary Fig.5A, we present the colormap of the bin counts obtained on a 200 nm long junction (not presented in the main text), as a function of the dc and rf drives I and I_{rf}. The measurement is taken at $f = 3\,\mathrm{GHz}$ and $800\,\mathrm{mK}$. For this frequency, we observe the complete suppression of the first Shapiro step. Again, the first oscillation is strongly disturbed as detailed in the main text. More surprisingly, a weak but distinct suppression of the third step $n = 3$ can be seen for low rf currents. It is emphasized by plotting the amplitude of each pair of steps (Supplementary Fig.5B). First, as described in the main text, the amplitude of the first step (red line) is fully suppressed up to the crossing point where the $n = 0$ region vanishes, while the second step (green line) is fully visible. Then, the amplitude of the $n = 3$ step (cyan line) is smaller than the amplitude of the $n = 4$ one (purple line) while the opposite is usually expected from RSJ models.

This could constitute evidence of a missing or suppressed third step, clearly observed only in one sample. However, other irregularities in the higher order steps are sometimes visible (see Fig.3 in the main article), so that this evidence has to be considered carefully.

D. Hysteresis, bias instability and Shapiro steps

In Supplementary Fig.6, we present two datasets measured on the same junction as the one presented in section C at two different temperatures. To emphasize the role of the control parameters (dc and rf currents I and I_{rf}), we now plot the differential resistance dV/dI as a colormap, as a function of these two parameters. On the left panel, measurements were obtained at $f = 3\,\mathrm{GHz}$ at the base temperature of the dilution refrigerator ($T = 12\,\mathrm{mK}$). On the right panel, measurements were obtained in the same conditions ($f = 3\,\mathrm{GHz}$) except for the temperature, here set high enough to suppress the hysteresis ($T \simeq 800\mathrm{mK}$).

Shapiro steps are identified as black regions where $dV/dI \simeq 0$, while blue lines between black regions emphasize transitions between the different plateaus. A simultaneous read-

ing of the voltage V gives access to the step index n (a few of them are indicated directly on Supplementary Fig.6). The dc current is swept in the direction indicated by the white arrow (from negative towards positive bias). At $T = 12\,\mathrm{mK}$, the very clear asymmetry at low rf excitations is a signature of the hysteresis observed in the I-V curve presented in the main text. The bistable dynamics that leads to hysteresis prevents the development of the phase-locked dynamics responsible for the Shapiro steps. Consequently, the latter are missing in the hysteretic region. In contrast, all steps are clearly visible at higher temperature ($T \simeq 800\,\mathrm{mK}$), except for the $n = 1$ step which is fully suppressed at $f = 3\,\mathrm{GHz}$.

It is possible to make a clear distinction between missing steps due to hysteresis and the missing $n = 1$ step attributed to a 4π-periodic supercurrent. First, as can be seen on Supplementary Fig.6, hysteretic switchings present vertical tangents (similar to ref.10) while the doubled Shapiro step always exhibits a finite slope. Second, hysteresis is characterized by an asymmetry depending on the sweep directions, which is not the case of the missing step in our measurements. Third, the anomalous splitting of the $n = 1/n = 2$ steps beyond the pinch-off of the supercurrent (corresponding to the dark fringe in the bin counts, discussed in the main text) that accompanies a missing $n = 1$ step remains visible at all temperature regardless of the presence of hysteresis for low rf powers. For these reasons, it appears clear that one can safely neglect hysteresis as the origin of the missing $n = 1$ step. To avoid any problem, most measurements were performed at a temperature high enough to suppress the hysteresis (typically 450 to 800 mK).

E. Shapiro steps on a shunted device

Furthermore, it is in fact possible to rule out bias instabilities as a possible mechanism for the missing $n = 1$ step. By adding a shunt resistance in parallel with the junction, one can indeed suppress hysteresis[11] and work in a configuration that approaches the voltage bias regime. To do so, we add a $10\,\Omega$ resistor in series with the junction, and shunt these two elements with a $1\,\Omega$ resistor. Thus, the current flowing through the junction

is accessed by measuring the voltage across the $10\,\Omega$ resistor, together with the voltage across the junction.

Results obtained at the base temperature of the fridge (12 mK) and with an rf excitation at 4.2 GHz are shown in Supplementary Fig.7. First, the black solid line in Supplementary Fig.7A represents the $I-V$ curve in the absence of rf irradiation. We do not detect in this configuration any instability. At low voltages, one sees that $dV/dI < 0$. In this region, a pure current bias generates bias instability and hysteresis, which is here suppressed by the shunt resistance. When the rf irradiation is switched on (colored plain lines), Shapiro steps become visible in the $I-V$ curves. As previously, the step $n = 1$ is clearly suppressed at low rf excitation (blue lines). Increasing the rf drive (from blue to yellow lines), one sees the $n = 1$ step is recovered at high drive amplitude as previously.

Supplementary Fig.7 B and C present the voltage histograms and differential resistance dI/dV as a function of the normalized DC voltage V and rf current I_{rf}. Shapiro steps are visible as previously as maxima following horizontal lines. As for the measurements shown in the main text, the first step $n = 1$ is fully suppressed up to the oscillating regions. Though the contrast is not as good, the "dark fringe" at finite voltage described in the main text is also visible.

F. Magnetic field dependence

Further investigations of the anomalous Shapiro response have been carried out in the presence of perpendicular-to-plane magnetic fields. First, when the I-V curve is measured without rf irradiation, a Fraunhofer-like diffraction pattern of the critical current is observed (plotted as a red line in Supplementary Fig.8, upper panel). In the junction presented here, the pattern is slightly distorted (probably due to flux-trapping in the magnet). The periodicity in the magnetic field has been evaluated from undistorted patterns in various other samples. It corresponds to a conventional periodicity as previously reported on similar samples[9], and as expected for ballistic systems with such aspect ratios[12,13].

The observation of Shapiro steps in the presence of the magnetic field reveals that the anomalous doubling of the first step is only observed when the critical current is high enough, namely only at the center of the central lobe ($B \lesssim 0.3\,\mathrm{mT}$) and to a lesser extent at the centers of the second and third lobe. The ratio Q_{12} of the first ($n = 1$) and second ($n = 2$) step widths (as introduced in the main text) is plotted as a function of the magnetic field in Supplementary Fig.8 (as a blue line, lower panel). The plot reproduces the variations observed in the critical current: the reduction of the first step is distinctly observed for high critical currents, yielding low values for Q_{12}, while Q_{12} increases for low critical currents near the minima of the Fraunhofer pattern. This behavior can be understood by assuming a constant value of the 4π-supercurrent fraction $I_{4\pi}/I_{\mathrm{c}}$: the frequency $f_{4\pi}$ rapidly decreases when I_{c} decreases, so that a correct observation of the anomalous step is not possible according to the criterion $f < f_{4\pi}$.

One could expect that a small magnetic field induces a splitting of the Andreev bound states. This would consequently weaken the effect of Landau-Zener transitions (see section C) and allow us to exclude or confirm Landau-Zener transitions as responsible for the anomalous Shapiro response. Given the large g-factor in HgTe (around $g \simeq 20$ for bulk HgTe), this effect could show up at relatively weak fields. To generate a splitting of $0.01\Delta_{\mathrm{i}}$ that significantly alters Landau-Zener transitions, a typical magnetic field B_0 is required, with $g\mu_B B_0 \simeq 0.01\Delta_{\mathrm{i}}$, yielding $B_0 \simeq 3.5\,\mathrm{mT}$. The presence of a week magnetic field does not seem to suppress the 4π-periodic contribution. It thus does not reveal any clear signature of energy splitting of the Andreev spectrum. This supports an interpretation based on the presence of a topological Andreev bound state. Indeed the latter is predicted to have no spin-degeneracy and therefore the level crossing at phase differences π, 3π, etc. should persist even in the presence of a magnetic field on the TI.

SUPPLEMENTARY NOTE 3 - ADDITIONAL RESULTS ON GRAPHENE-BASED JUNCTIONS

A. Typical properties of the graphene-based junctions

In this section, we briefly present measurements of the Shapiro response of graphene-based devices. First, graphene flakes on hexagonal Boron-Nitride are fabricated by a van der Waals stacking method. Superconducting contacts are patterned by standard electron beam lithography and Niobium is deposited by magnetron sputtering. The geometry of the presented devices is similar to the one used for the HgTe devices, with the graphene weak link width ranging between 1.5 µm and 2.5 µm, and the length between 200 nm and 300 nm.

The graphene flake exhibit densities between $-2.5 \times 10^{12}\,\mathrm{cm}^{-2}$ and $2.5 \times 10^{12}\,\mathrm{cm}^{-2}$, which can be tuned by the means of a back-gate. In the left panel of Supplementary Fig.11, we present the extracted normal state resistance R_n of the device as a function of the back-gate voltage V_g. The mobility approaches 3000 - $8000\,\mathrm{cm}^2\,\mathrm{V}^{-1}\,\mathrm{s}^{-1}$. Close to the Dirac point, this corresponds to a mean free path of around 50 nm. Consequently, these devices are not in the ballistic limit but are relatively close to it.

In the right panel of Supplementary Fig.11, we present the differential resistance dV/dI of one junction as a colormap, as a function of the back-gate voltage V_g and dc drive current I. The supercurrent then appears as a dark region ($dV/dI \simeq 0$), while the normal state exhibits finite values of dV/dI. The devices show a supercurrent over the whole back-gate range, yielding supercurrents of a few tens of nA around the Dirac point and up to 1.8 µA for large gate voltages.

B. Shapiro response of the graphene-based junctions

In this section we present two typical sets of data taken on the graphene-based junctions (see Supplementary Fig.12). The differential conductance dI/dV is plotted as a colorscale,

as a function of the dc voltage V and rf current drive I_{rf} for two frequencies ($f = 7\,\mathrm{GHz}$ and $5.5\,\mathrm{GHz}$). Despite fluctuations in the measurements (originating in fluctuations of the offset of one of our amplifiers), clear Shapiro steps are visible at the expected voltages $V_n = nhf/2e$. As frequency is lowered, the steps become less discernible but no sign of a vanishing $n = 1$ step is observed in these sets.

Measurements have been carried out for different values of the back-gate voltage (Dirac point, high n or p doping) and down to $4\,\mathrm{GHz}$ (for which the steps are hardly visible) but no sign of a missing $n = 1$ step has ever been spotted. Since no 4π-periodic modes are expected in the graphene junction, the crossover frequency is expected to be $f_{4\pi} = 0$ in this system. These measurements establish an experimental upper boundary $f_{4\pi} \ll 4\,\mathrm{GHz}$. Given the characteristic Josephson frequency of the device $f_{\mathrm{J}} \simeq 72\,\mathrm{GHz}$, this sets an upper boundary of $I_{4\pi} \ll 75\,\mathrm{nA}$.

SUPPLEMENTARY NOTE 4 : ELEMENTS OF MODELING

A. Standard RSJ model with a 2π-periodic supercurrent

The results shown here are obtained using the RSJ model presented in Methods, with a 2π-periodic sinusoidal current-phase relation $I_S(\phi) = I_{\mathrm{c}} \sin\phi$. The results obtained by Russer[14] are reproduced. The global visual agreement with our measurements is good, except the missing first step not accounted for in the standard RSJ model. In particular, the increasing frequency of oscillations (with respect to the rf current needed for the pinch-off of the supercurrent) is clearly observed.

In the lower panels, the step amplitudes for $n = 0, 1, 2, 3, 4$ are plotted as a function of the rf current amplitude I_{rf}. Again, one observes the effect of the excitation frequency f. As f decreases, the width of the first lobe gets much larger than the widths of the other lobes. Moreover, these graphs also show that the step amplitudes (hence their visibility) decrease (with respect to the critical current). This phenomenon, clearly observable in our measurements (lower panels of Fig.3, main text), limits our measurements to $f \geq 2\,\mathrm{GHz}$.

B. Extended RSJ model with a 4π-periodic contribution:

Simulations have also been carried out when adding a 4π-periodic contribution following Dominguez *et al.*[15]. We present in this section our results : the appearance of the doubled step is qualitatively well described as in the previous reference, but a quantitative agreement has not been obtained yet. The results are presented below in the following manner. First, we show that the addition of a 4π-periodic contribution $I_{4\pi} \sin\phi/2$ to a sinusoidal 2π-periodic current-phase relation (CPR) $I_{2\pi} \sin\phi$ is responsible for the disappearance of all odd steps at low frequency $f < f_{4\pi}$, in a comparable way to what we experimentally observe on the first step $n = 1$. Then the marginal effect of the 2π-periodic CPR is illustrated by comparing I-V curves in the presence of rf irradiation for a few different CPRs. No generic 2π-periodic CPR is found to show missing odd steps, while the addition of a small 4π-periodic term enforces the disappearance of odd steps regardless of the 2π-periodic term.

a. Effect of frequency on a $2\pi + 4\pi$-periodic supercurrent: We simulate the effect of a small 4π-periodic contribution $I_{4\pi} \sin\phi/2$. In Supplementary Fig.14, we show the effect of frequency on a CPR of the form $I_{4\pi} \sin\phi/2 + I_{2\pi} \sin\phi$ by plotting for different frequencies the histograms of the voltage as function of the rf current I_{rf} (upper panels) and the amplitudes of steps $n = 0$ to $n = 4$ (lower panels). The parameters are $I_{2\pi} = 1$, $I_{4\pi} = 0.15$, so that $f_{4\pi} = 0.15 f_{\mathrm{J}}$. For a high frequency $f > f_{4\pi}$ (left, $f = 0.5 f_{\mathrm{J}}$), all steps are visible, and the 2D plot is similar to the one obtained without 4π-periodic contribution. As frequency is lowered (center, $f = f_{4\pi} = 0.15 f_{\mathrm{J}}$, and right, $f = 0.05 f_{\mathrm{J}}$), the amplitude of odd steps progressively decreases, and these odd steps are completely suppressed at low power. The simulated behavior of the $n = 1$ step is very similar to the one experimentally observed, and thus reinforces our interpretation. Though the crossover is not very sharp, it happens for $f = f_{4\pi}$ ($= 0.15 f_{\mathrm{J}}$ in the case of Supplementary Fig.14). One also observes that the oscillatory pattern (at high power) is also progressively modified from a 2π- to a 4π-dominated pattern. In particular, odd steps show a very pronounced first minimum. The dark fringe we experimentally observe in the oscillatory pattern is understood as

the result of the progressive towards a pattern with a halved number of oscillations, thus yielding progressively suppressed lobes.

b. Effect of the CPR Though the presence of a small 4π-periodic contribution $I_{4\pi}\sin\phi/2$ is found necessary to observe vanishing odd steps, the exact description of the 2π-periodic supercurrent does not influence much the Shapiro response. To illustrate this finding, we focus on three different CPR. The first one is a standard $I_{2\pi}\sin\phi$ contribution (obtained for a tunnel junction for example), as in the previous paragraph. For the other two, we assume that the current is carried as a single mode of transmission $\tau = 0.8$ and 0.99 respectively. The skewness of the CPR increases with transmission. The current carried by such a mode is then given by $\tau\sin\phi/\sqrt{1-\tau\sin^2\phi/2}$. We normalize all CPRs such that $I_\mathrm{c} = I_{2\pi} = 1$. Supplementary Fig.15 shows in the upper panels the CPR and in the lower panels an I-V curve in the presence of rf irradiation, for a low frequency $f = 0.05 f_\mathrm{J} = f_{4\pi}/3$. On the left side, the CPR contains only this 2π-periodic supercurrent, and the obtained I-V curves exhibits all integer steps and do not depend on the exact CPR. When a 4π-periodic supercurrent is added, all odd steps are suppressed or reduced. The CPR matters marginally, and only seem to control the exact detail of the transition from the 2π- to the 4π-dominated regime : the transition is slightly faster for a skewed CPR.

C. Evaluation of Landau-Zener probabilities

If some Andreev bound states have very high transmission (or small energy gap δ, see Supplementary Fig.16), non-adiabatic Landau-Zener processes may be responsible for an additional 4π-periodic contribution to the supercurrent. Namely, some gapped 2π-periodic Andreev levels would behave as effective 4π-periodic modes, in the absence of true 4π-periodic modes. To our knowledge, studies of the effect of Landau-Zener transitions on the dynamics of Josephson junctions are scarce[15–19] in general and systems with multiple Andreev levels have not been explored yet. We thus use a single mode approximation[15], and crudely assume that one level (with lowest gap δ) has a predominant role in Landau-

Zener processes.

Following Dominguez *et al.*[15], we include in our simulations stochastic Landau-Zener transitions at the anticrossings, with a probability P, and partially reproduce their results. The exact motion of the phase difference is hard to picture, but the general trend can be understood on a heuristic basis. The phase will undergo shifts of 4π (per period of the drive) in the case of a Landau-Zener transition and only 2π in the absence of transitions. On average this results in a non-universal Shapiro step that is neither $hf/2e$ nor hf/e. These two values are recovered for $P = 0$ and $P = 1$ respectively. The results of our simulations are presented in Supplementary Fig.17A, in which a close-up of the $n = 2$ Shapiro step is presented, for different values of P. As Dominguez *et al.*, we observe a splitting of the step for P close to 1, with voltage plateaus that deviate from the quantized value hf/e until the split steps are eventually less discernible for $P \lesssim 0.7$. Our experimental results do not show any splitting or deviation to the quantized value, with an accuracy of a few percents. This indicates that the probability must be $P > 0.97$ ($P = 1$ being equivalent to having a fully gapless mode).

From the previous model, one can estimate the importance of the Landau-Zener transitions. First, we solve the RSJ equation, and obtain the phase $\phi(t)$ and its derivative $\dot{\phi}(t) \propto V(t)$ as a function of time t. The parameters are chosen such that the junction lies on the first Shapiro step ($V = \langle V(t) \rangle = \frac{hf}{2e}$). In that case, a high probability of Landau-Zener transitions would lead the junction to exhibit a doubled step.

A typical plot of $\phi(t)$ and $V(t)$ is shown in Supplementary Fig.17B, with $\phi(t)$ as a red line and $V(t) \propto \dot{\phi}(t)$ as a blue line for the following parameters: $I = 0.5\, I_{\rm c}$, $I_{\rm rf} = 0.8\, I_{\rm c}$, $f = 0.2\, f_{\rm J}$. One first observes that the phase ϕ follows an anharmonic motion synchronized with the excitation drive at frequency f: during one period of duration $1/f$, the phase ϕ increases by 2π, yielding an averaged voltage $V = hf/2e$ as expected for the first Shapiro step. Equivalently, one can calculate the average of $V(t)$ and obtain $V = \langle V(t) \rangle = \frac{hf}{2e}$. Then, we access the time t for which ϕ reaches the anticrossing (for $\phi(t) = 3\pi$ for example) and read the derivative of the phase $\dot{\phi}|_{3\pi}$ at this point or equivalently the voltage $V|_{3\pi}$.

Finally, the Landau-Zener probability P is obtained from the following equation[15]:

$$P = \exp\Big(-2\pi \frac{\delta^2}{\Delta_{\mathrm{i}} \hbar \dot{\phi}} \Big)$$

This assumes a generic pair of Andreev bound state with energy $\varepsilon_{\pm}(\phi) = \pm\sqrt{\delta^2 + \Delta_{\mathrm{i}}^2 \cos^2 \phi/2}$.

For the graphs presented here, we obtain $V|_{3\pi} \simeq 6.4 \frac{hf}{2e}$. This yields a probability $P > 0.98$ if $\delta < 6\,\mu\mathrm{eV}$. The most stringent constraint is obtained for the lowest frequencies accessible in the experiment (2 GHz). With the criterion $P > 0.97$, we obtain $\delta \lesssim 9\,\mu\mathrm{eV}$ for $\Delta_{\mathrm{i}} = 0.12\,\mathrm{meV}$ ($L = 400$ and $600\,\mathrm{nm}$), and $\delta \lesssim 18\,\mu\mathrm{eV}$ for $\Delta_{\mathrm{i}} = 0.35\,\mathrm{meV}$ ($L = 150\,\mathrm{nm}$). This corresponds to a transmission $\sqrt{1 - (\delta/\Delta_{\mathrm{i}})^2} \geq 0.994$ in both cases.

SUPPLEMENTARY REFERENCES

1 G. Tkachov and E.M. Hankiewicz. Helical Andreev bound states and superconducting Klein tunneling in topological insulator Josephson junctions. *Physical Review B*, 88:075401, 2013.

2 G.E. Blonder, M. Tinkham, and T.M. Klapwijk. Transition from metallic to tunneling regimes in superconducting microconstrictions: Excess current, charge imbalance, and supercurrent conversion. *Physical Review B*, 25:4515–4532, 1982.

3 W.L. McMillan. Tunneling model of the superconducting proximity effect. *Physical Review*, 175:537–542, 1968.

4 I. Sochnikov, L. Maier, C.A. Watson, J.R. Kirtley, C. Gould, G. Tkachov, E.M. Hankiewicz, C. Brüne, H. Buhmann, L.W. Molenkamp, and K.A. Moler. Nonsinusoidal Current-Phase Relationship in Josephson Junctions from the 3D Topological Insulator HgTe. *Physical Review Letters*, 114, 2015.

5 G. Fagas, G. Tkachov, A. Pfund, and K. Richter. Geometrical enhancement of the proximity effect in quantum wires with extended superconducting tunnel contacts. *Physical Review B*, 71:224510, 2005.

[6] N.B. Kopnin, A.S. Mel'nikov, I.A. Sadovskyy, and V.M. Vinokur. Weak links in proximity-superconducting two-dimensional electron systems. *Physical Review B*, 89:081402, 2014.

[7] A. Valizadeh, M.R. Kolahchi, and J.P. Straley. On the Origin of Fractional Shapiro Steps in Systems of Josephson Junctions with Few Degrees of Freedom. *Journal of Nonlinear Mathematical Physics*, 15:407–416, 2008.

[8] D.E. McCumber. Effect of ac Impedance on dc Voltage-Current Characteristics of Superconductor Weak-Link Junctions. *Journal of Applied Physics*, 39:3113, 1968.

[9] J.B. Oostinga, L. Maier, P. Schüffelgen, D. Knott, C. Ames, C. Brüne, G. Tkachov, H. Buhmann, and L.W. Molenkamp. Josephson Supercurrent through the Topological Surface States of Strained Bulk HgTe. *Physical Review X*, 3:021007, 2013.

[10] H. Courtois, M. Meschke, J. Peltonen, and J. Pekola. Origin of Hysteresis in a Proximity Josephson Junction. *Physical Review Letters*, 101:067002, 2008.

[11] M. Chauvin. *The Josephson Effect in Atomic Contacts*. PhD thesis, Univeristé Pierre et Marie Curie - Paris 6, 2005.

[12] J.P. Heida, B.J. van Wees, T.M. Klapwijk, and G. Borghs. Nonlocal supercurrent in mesoscopic Josephson junctions. *Physical Review B*, 57:R5618–R5621, 1998.

[13] U. Ledermann, A. Fauchère, and G. Blatter. Nonlocality in mesoscopic Josephson junctions with strip geometry. *Physical Review B*, 59:R9027–R9030, 1999.

[14] P. Russer. Influence of Microwave Radiation on Current-Voltage Characteristic of Superconducting Weak Links. *Journal of Applied Physics*, 43:2008, 1972.

[15] F. Domínguez, F. Hassler, and G. Platero. Dynamical detection of Majorana fermions in current-biased nanowires. *Physical Review B*, 86:140503, 2012.

[16] D. Averin and A. Bardas. ac Josephson Effect in a Single Quantum Channel. *Physical Review Letters*, 75:1831–1834, 1995.

[17] M.F. Goffman, R. Cron, A. Levy Yeyati, P. Joyez, M.H. Devoret, D. Esteve, and C. Urbina. Supercurrent in atomic point contacts and Andreev states. *Physical Review Letters*, 85:170–173, 2000.

[18] M. Chauvin, P. vom Stein, D. Esteve, C. Urbina, J. Cuevas, and A. Yeyati. Crossover from

Josephson to Multiple Andreev Reflection Currents in Atomic Contacts. *Physical Review Letters*, 99:067008, 2007.

[19] J.D. Sau, E. Berg, and B.I. Halperin. *Preprint at http://arxiv.org/abs/1206.4596 (2012).*
